# Biological and prognostic insights into the prostaglandin D2 signaling axis in lung adenocarcinoma

**DOI:** 10.3389/fphar.2025.1562261

**Published:** 2025-05-22

**Authors:** Qiang Liu, Huiguo Chen, Dongfang Tang, Huibiao Zhang, Shaogeng Chen, Yiran Meng, Boying Zheng, Fei Liu, Jing Zhou, Wen Zhang

**Affiliations:** ^1^ Department of Thoracic Surgery, Peking University International Hospital, Beijing, China; ^2^ Department of Cardiothoracic Surgery, The Third Affiliated Hospital of Sun Yat - sen University, Guangzhou, Guangdong, China; ^3^ Department of Thoracic Surgery, Huadong Hospital Affiliated to Fudan University, Shanghai, China; ^4^ Department of Thoracic Surgery, Quanzhou First Hospital Affiliated to Fujian Medical University, Quanzhou, Fujian, China; ^5^ Hangzhou Astrocyte Technology Co,. Ltd, Hangzhou, China; ^6^ Department of Thoracic Surgery, Fuyang People’s Hospital, Fuyang, Anhui, China; ^7^ Department of Thoracic Surgery, Fourth Medical Center of PLA General Hospital, Beijing, China

**Keywords:** metabolic signaling, lung adenocarcinoma, prostaglandin D2, CX3CR1+ NK/t cells, prognostic model

## Abstract

**Background:**

Tumor metabolism reprogramming is a hallmark of cancer, but metabolite-mediated intercellular communication remains poorly understood. To address this gap, we estimated and explored communication events exploring based on single‐cell RNA data, to explore the metabolic landscape of tumor microenvironment (TME) in lung adenocarcinoma (LUAD) and identify novel metabolite signaling axis.

**Methods:**

The scRNA-seq dataset was subjected to dimensionality reduction using the Seurat package. Cell annotation was manually performed using typical markers from Cell Marker 2.0 and previous studies. Single‐cell metabolite abundance and communication events were inferred using MEBOCOST. The TCGA‐LUAD datasets was used to estimate and analyze immune cell infiltration levels and tumor hot score using the ESTIMATE and ssGSEA algorithms. Additionally, survival analysis was conducted on genes within relative signaling axis. All analysis above in TCGA‐LUAD dataset was validated by two Gene Expression Omnibus (GEO) datasets. The expression patterns of PTGDR and PTGDS were validated by RT‐qPCR and fluorescence in situ hybridisation.

**Results:**

Five landmark metabolites across cell types were identified as prostaglandin D2 (PGD2), D-Mannose, Choline, L-Cysteine, and Cholesterol of TME in LUAD. Prostaglandin D2 (PGD2) emerged as a key player, primarily produced by fibroblasts and plasmacytoid dendritic cells (pDCs) by via the *PTGDS* gene and by mast cells via the *HPGDS* gene. PGD2 signaling was shown to primarily be received by the PGD2 receptor (*PTGDR*) on NK/T cells and transported by the *SLCO2A1* transporter on endothelial cells. *CX3CR1+* NK/T cells, which are prominent cytotoxic populations, as a PGD2 autocrine signaling axis, are involved in PGD2 autocrine signaling, while KLRC2+ NK, DNAJB1+ NK cells and CD8+ MAIT cells participate in PGD2 paracrine signaling. PGD2 may also assist lactate efflux via *SLCO2A1* on endothelial cells. The clinical relevance of the PGD2 signaling axis was validated across multiple bulk RNA datasets, showing that it is associated with the infiltration of above immune cells such as DNAJB1+ NK cells, and linked to better prognosis in LUAD. Furthermore, we found that a risk model developed based on this signaling axis could predict responses to immune therapy in hot and cold tumors, suggesting potential drugs that may benefit low-risk patients. These findings were further supported by RT-qPCR and immunofluorescence data, which confirmed the downregulation of PTGDS and PTGDR in LUAD tumor tissues compared to normal tissues.

**Conclusion:**

Collectively, these results suggest that PGD2 and its signaling axis play a significant role in tumor-suppressive and anti‐inflammatory effects in LUAD, with potential applications in prognosis management and therapy decision‐making.

## Introduction

Over the past 2 decades, reprogramming of tumor metabolism within the tumor microenvironment (TME) and its role in the development of cancer therapies have garnered significant attention (Mao et al., 2024; Nong et al., 2023; Park et al., 2020). Additionally, exploring the connections between cellular metabolism, cancer, and the immune system has led to an understanding of tumor metabolism, facilitating the development of metabolite-based therapies. Actually, many metabolites and can be released into the extracellular where they enter other cells to exert their effects, facilitating intercellular crosstalk (Wang and Lei, 2018; Liu et al., 2014; Mao et al., 2024). For example, cancer-associated fibroblasts (CAFs) play a critical role in pancreatic cancer metabolism by secreting cysteine, a process mediated by the TGF-β/SMAD3/ATF4 signaling axis (Zhu et al., 2024). However, due to technical limitations, most mechanisms through which metabolites mediate intercellular interactions and their roles whin TME remain underexplored. In recent years, advancements in metabolism inference algorithms based on single-cell transcriptomic data have provided new opportunities to address these gaps (Alghamdi et al., 2021; Nathaniel et al., 2023; Wagner et al., 2021; Zheng et al., 2022). Among them, MEBOCOST contains a large amount of metabolite data from the fluid environment (Zheng et al., 2022).

Lung cancer is the most frequently diagnosed cancer,accounting for one in eight cancers worldwide (Bray et al., 2024). Lung adenocarcinoma (LUAD) is the most prevalent histological type of Lung cancer (Chen et al., 2014). The discovery of metabolic markers in lung cancer, particularly LUAD, is gaining interest, though research is often limited by small sample sizes (Klupczynska-Gabryszak et al., 2024; Moreno et al., 2018; Zhao et al., 2020; Nie et al., 2021). A systematic review has reproted that more than 150 metabolites are linked to altered lung cancer metabolism (Madama et al., 2021). However, the clinical application of these potential biomarkers is limited due to a high rate of false positives and insufficient understanding of disease mechanisms. Although extensive research has been conducted on LUAD prognosis in relation to histology, mutations, gene expression, proteomics, and the microbiome, studies focusing on metabolites and intercellular metabolic signaling axes remain scarce.

**FIGURE 1:**
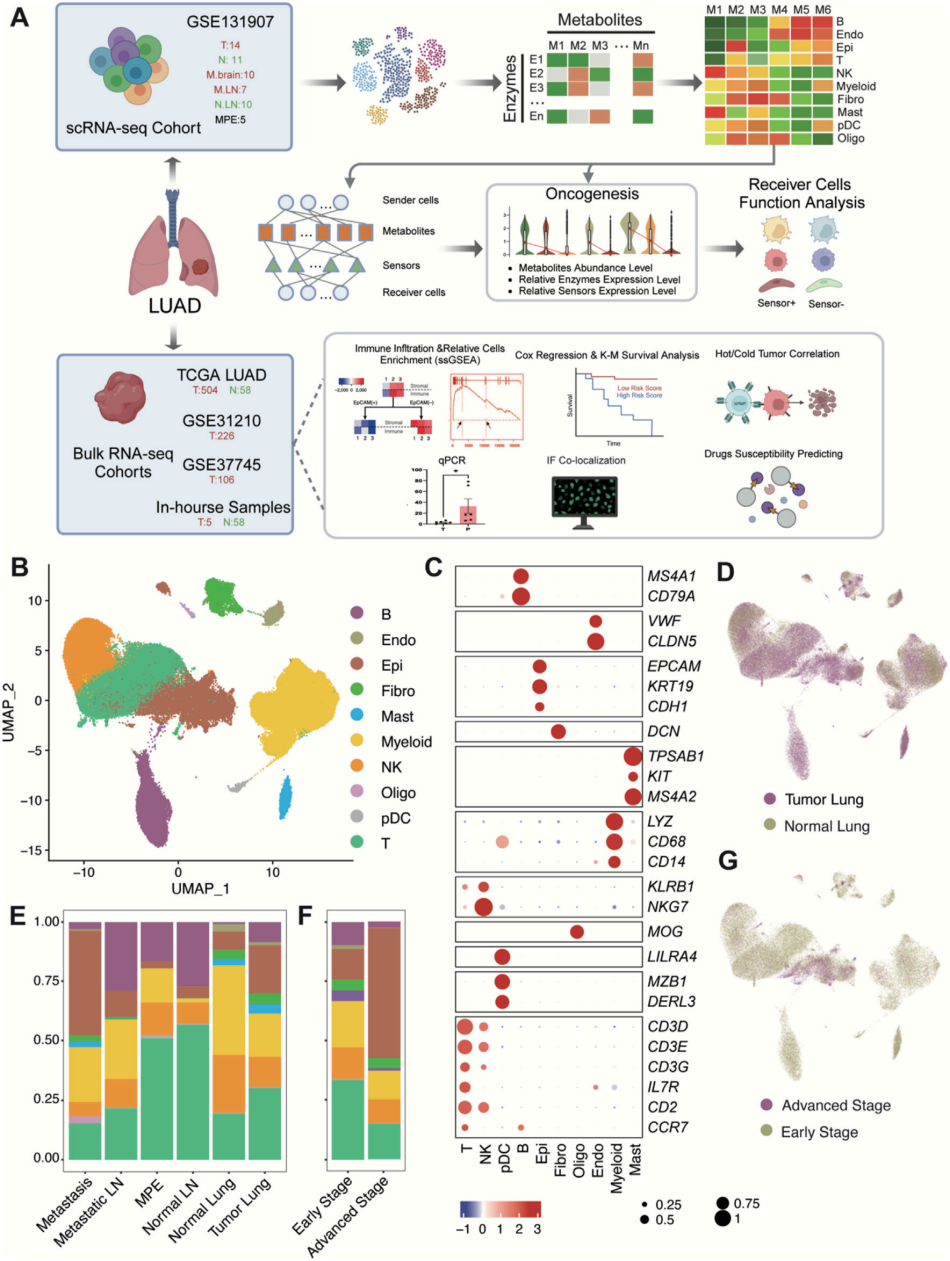
Schematic workflow diagram of this study and single-cell transcriptome atlas of LUAD. **(A)** Workflow diagram. **(B)** Uniform manifold approximation and projection (UMAP) plot of 177,042 cells colored by cell type. Raw grouped clusters were presented in Supplementary Figure S1C. **(C)** Dot plot for the expression pattern of marker genes of major cell type. The intensity of color represents Expression (Z-score normalized mean expression level); the dot size indicates the percentage of the respective cell type. **(D)** UMAP plot of 177,042 cells colored by lung tumor and normal tissues. **(E)** Overview of cell type composition across various tissue locations. **(F)** Overview of cell type composition across different stages (Early stage include stage I-III, advanced stage is stage IV). The sample proportions of each cell type and the cellular composition of each sample are shown in Supplementary Figure S2. **(G)** UMAP plot of 177,042 cells colored by early stage and advanced stage. **(B)** B cells; Endo: endothelial cells; Epi: epithelial cells; Fibro: fibroblasts; Mast: mast cells; Myeloid: myeloid cells (excluding pDC and Mast); NK: Natural Killer cells; Oligo: Oligodendrocytes; pDC: Plasmacytoid dendritic cells; T: T cells.

In this study, we collected a single-cell RNA sequencing (scRNA-seq) dataset of baseline LUAD to investigate metabolite abundance and infer their crosstalk events (Figure 1A). Among the identified metabolites, prostaglandin D2 (PGD2) emerged as a key player in the tumor microenvironment (TME) of LUAD, actively mediating intercellular communication. We identified the main sender cells of PGD2 and its receiver cells, uncovering its anti-tumor effects through the activation of T cells and NK cells via the PGD2 receptor (*PGDR*). Simultaneously, we validated the mRNA and protein expression patterns of these key genes in retrospectively collected samples. This mechanism was further validated across three bulk RNA-seq datasets, highlighting the potential clinical relevance of the PGD2-associated metabolic signaling axis. Our findings not only elucidate the functional characteristics of PGD2 in LUAD but also provide a novel perspective on exploring metabolic biomarkers.

## Methods

### Data collection

The scRNA-seq dataset GSE131907 (Kim et al., 2020) was download from GEO database, containing 208,506 immune cells after the quality measures so as to remove less informative cells. Of which15 tumor tissues, 11 distant normal lung, 10 normal lymph node, 10 metastatic brain tissue and seven metastatic lymph node samples from 44 LUAD patients with treatment-naïve (58 samples). Additionally five pleural fluid samples were obtained from LUAD patients with malignant pleural effusion. After further quality control, the sample EBUS_49 (GSM3827138) from an advanced-stage patient was removed due to significant red blood cell contamination. We also filtered out cells based on the following criteria: Reads Count <500, Feature Numbers <200, Mean unique molecular Identifier (UMI) count per genes <0.8, Mitochondrial gene percentage >10% and Ribosomal gene percentage >60%. Additionally, potential doublets were excluded using DoubletFinder with a DoubletRatePer threshold of 0.005. After filtering, the remaining high-quality cells count for GSE131907 was 185,197, which were used for subsequent analysis.

A total of three independent bulk RNA LUAD datasets were collected for this study. Standardized RNA-seq expression data (TPM matrix) comprising 562 samples, including 504 tumor samples and 58 distant normal lung samples, were obtained from TCGA (https://portal.gdc.cancer.gov/). Additionally, two microarray datasets, GSE31210 (225 LUAD tumor samples) and GSE37745 (106 LUAD tumor samples), were sourced from the GEO database (https://www.ncbi.nlm.nih.gov/geo/). The microarray datasets were preprocessed by mapping probe IDs to gene symbols based on the corresponding microarray chip platforms. For genes represented by multiple probes, the mean value of all probes was used.

### Clustering and major cell type identification in scRNA-seq data

All analyses were conducted using the Seurat v5.1.0 package (Hao et al., 2024). Initially, NormalizeData was applied with method = “LogNormalize” to normalize read counts. Cell cycle scoring was performed using the CellCycleScoring function with 43 S phase and 54 G2M phase genes (Supplementary Table S1), estimate each cell’s S.Score, G2M.Score, Phase and CC.Difference = S.Score - G2M.Score. Variable features were identified using the FindVariableFeatures function (method = vst, nfeatures = 2000) and filtered to exclude mitochondrial genes, hemoglobin genes, ribosomal protein genes, immunoglobulin genes, T-cell receptor genes, and AC233755.1. Data scaling was performed using the ScaleData function with features from variable features and vars.to.regress set to nCount_RNA, nFeature_RNA, and CC.Difference. Principal component analysis (PCA) was conducted (RunPCA, npcs = 30) for unsupervised clustering. To address batch effects, samples were integrated using the RunHarmony function from the harmony v1.2.1 package with default parameters (Korsunsky et al., 2019). Subsequently, neighbors were identified using FindNeighbors (reduction = “harmony”, n.neighbors = 40, min.dist = 0.5, dims = 1:30), and clusters were determined using FindClusters at a resolution 0.1. After reviewing the UMAP plot, 8,155 cells that mixed with epithelial clusters but expressed *CD3D* and not *EPCAM* were removed. Finally, 177,042 remaining cells were reanalyzed following the steps above, resulting in the final valid clustering (Supplementary Figure S1).

The cell type of each cluster was defined based on recognized cell marker genes from previous studies and the CellMarker 2.0 database (Supplementary Figure S2A). Clusters one and five were identified as Myeloid cells based on the expression of *LYZ*, *CD68*, and CD14. Cluster three was classified as NK cells expressing *KLRB1* and *NKG7*, while cluster four was identified as B cells with markers *MS4A1* and *CD79A*. Cluster six was designated as Fibroblasts expressing *DCN*, and cluster 7 as Mast cells with markers *TPSAB1*, *KIT*, and *MS3A2*. Cluster eight was defined as Endothelial cells based on *VWF* and *CLDN5*, and cluster 9 as pDCs expressing *MZB1*, *DERL3*, and *LILRA4*. Cluster 10 was identified as Epithelial cells with markers *EPCAM*, *KRT19*, and *CDH1*, and cluster 11 as Oligodendrocytes expressing *MOG*. Finally, clusters 0, 12, 13, and 14 were identified as T cells based on the expression of *CD3D*, *CD3E*, *CD3G*, *IL7R*, *CD2*, and *CCR7*.

### Metabolic cell-cell communication analysis by MEBOCOST

To estimate metabolic communication stress, we downsampled major cell types exceeding 6,000 cells to 6,000 randomly selected cells, creating a metabolite estimation object (Supplementary Table S2). Using the infer_commu function in MEBOCOST v1.0.4 (Zheng et al., 2022), we set cutoff_exp and cutoff_met to 0 (retaining all nonzero metabolites or sensors) and cutoff_prop to 0.01 (at least 1% of cells expressing the sensor or presenting the metabolite in the specified cell type). Communication events were assessed for significance using permutation_test_fdr, retaining events with pval_cutoff = 0.9 based on the default database configuration (Supplementary Table S3).

To evaluate differences in metabolite abundance among cell types, we created a Seurat object from the estimated metabolite abundance matrix. Cells were clustered by selecting the top 200 features using FindVariableFeatures and applying unsupervised clustering (RunPCA, FindNeighbors, FindClusters) with parameters consistent with prior clustering in the scRNA-seq data analyses.

**FIGURE 2:**
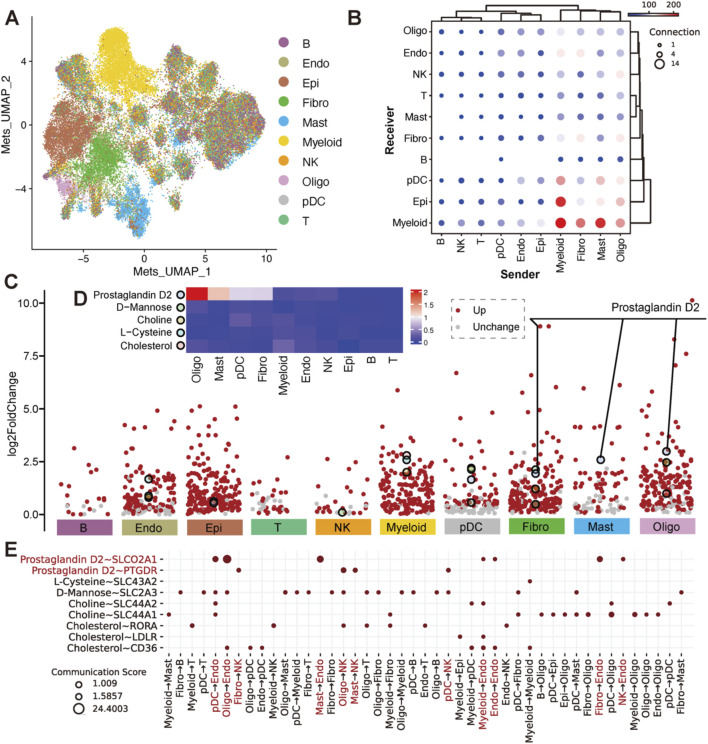
Estimation of metabolites and their differential abundance with sensor expression across cell types. **(A)** UMAP plot of cells based on top 200 variable metabolite abundance, colored by cell type. **(B)** Dot plot of communications between cell types in pairwise, where x-axis was the sender cell types, the y-axis was the receiver cell types. The dot size indicated the number of metabolite-sensor communications between a sender and a receiver. The color of the dot represented the overall confidence of communications between the sender and receiver. Only metabolite communication scores >1 and metabolite proportions in senders and sensor proportions in receivers >0.1 are shown. **(C)** Volcano plots of differential metabolites (log2FoldChange >0) across 10 cell types. Metabolites with Wilcoxon test p.adjust <0.001 are shown in red. The top 20 metabolites with the highest fold change in each cell type (also appearing in the events of **(B)** are highlighted with larger, differently colored circles. Details was in Supplementary Table S4. **(D)** Heatmap of the top differential metabolites. Colors represent the mean abundance level in each cell type. **(E)** Dot plot of metabolite-sensor communications for differential metabolites (Prostaglandin D2, L-Cysteine, D-Mannose, Choline and Cholesterol). Rows represent metabolite-sensor pairs, columns represent sender cell types. Prostaglandin D2 is highlighted in red.

Communication Results were visualized using custom thresholds (pval_cutoff = 0.01, comm_score_cutoff = 1, cutoff_prop = 0.1 for sender and receiver sensors/metabolites, details in Supplementary Table S4). The top 20 metabolites for each cell type were identified by log2FoldChange >0 and p.adjust <0.001 (Wilcoxon test) with comm_score >1 (Supplementary Table S5). Considering unknown sensors for some metabolites, only top metabolites associated with detectable communication events were labeled, as shown in Figure 2C.

### Re-clustering of NK and T cells

We extracted 24,489 NK cells and 56,674 T cells for further analysis. The analysis followed similar methods as prior clustering in the scRNA-seq data. VariableFeatures (nfeatures = 2000) were selected, followed by scaling, PCA (npcs = 30), and clustering as previously described. For NK cell clustering, the top 30 PCs were selected with a resolution parameter of 0.4 and min.dist = 0.3. we first filtered out 6,146 cells that were *CD3D* negative but expressed EPCAM, which were considered contamination. Subsequently, for the remaining 50,528 cells, the top 15 PCs were selected with a resolution parameter of 0.4 and min.dist = 0.3.

We annotated 13 NK cell clusters based on *PTGDR* and *PTGDS* gene expression as well as referenced markers from previous studies (Tang et al., 2023). Clusters one was identified as CX3CR1+ NK based on the expression of *CX3CR1*. Cluster eight was classified as DNAJB1+ NK highly expressing *DNAJB1*, while cluster 0, 5, 7, 10,11 were identified as KLRC2+ NK with marker *KLRC2*. Clusters 2, 3, and six were designated as RGS1+ NK, marked by *RGS1*. Due to the lack of distinct reference markers, Cluster nine was designated as Unknown NK1; Clusters 4 and 13 as Unknown NK2; and Cluster 12 as Unknown NK3.

Using T cell markers (*CD8A* and *CD4*), we identified T cells and revealed two major populations. The CD8+ T cells were further divided into three clusters: CD8+ T1, characterized by MAIT markers (*SLC4A10*, *ZBTB16*, *NCR3*, *RORA*, and *KLRB1*); CD8+ T2, marked by naïve T cell markers (*CCR7*, *SELL*, *TCF7* and *LEF1*); and CD8+ T3, identified by cytotoxic markers (*GZMH*, *GNLY*, *GZMK*, *NKG7* and *GZMA*). Recent studies have also provided additional markers for CD8+ T cells, including *KIR2DL4*, *CX3CR1*, *TYROBP*, *CXCR5*, *CD69*, *RPS12*, *CD52* and *NME1*, which help in further characterizing CD8+ T cell populations and their functional states (Zheng et al., 2021).

All feature plots with gene expression density were greated using the plot_density function from the Nebulosa v1.14.0 package (Alquicira-Hernandez and Powell, 2021).

### Validation expression of genes in PGD2 signaling axis

To validate the involvement of PGD2 signaling components, we utilized several publicly available integrated datasets derived from pan - cancer studies. These datasets consolidate single-cell data from multiple studies, focusing on major cell lineages such as fibroblast cells (Gao et al., 2024), myeloid cells (Cheng et al., 2021), NK cells (Tang et al., 2023), and T cells (Zheng et al., 2021). For this analysis, we specifically extracted data related to LUAD or lung tissue, including normal samples.

The integrated fibroblast dataset (http://pan-fib.cancer-pku.cn) was used to investigate *PTGDS* expression, identifying its predominant cell subtypes and tissue localization. For myeloid cells, including pDCs and mast cells, the myeloid dataset (http://panmyeloid.cancer-pku.cn) provided information on the distribution of *PTGDS* and *HPGDS* across various lineages and tissues. The NK cell dataset facilitated the examination of *PTGDS* and *PTGDR* in the context of PGD2 signaling reception. Lastly, the T-cell dataset (http://cancer-pku.cn:3838/PanC_T) offered detailed insights into *PTGDS* and *PTGDR* expression specifically in CD8^+^ T cells. These datasets collectively facilitated a detailed cross-validation of PGD2 signaling components in various cell types and biological contexts.

### Definition of gene sets for signature calculation

To evaluate the functional variations between SLCO2A1^+/−^Endo cells, PTGDS^+/−^NK cells, PTGDR^+/−^NK cells, and PTGDR^+/−^T cells, we defined L-lactate dehydrogenase, cytotoxicity, inflammatory and stress-related gene sets after comprehensive compilation of previous studies (Tang et al., 2023). The L-lactate dehydrogenase gene sets were defined as *LDHA* and *LDHB*; The cytotoxicity gene sets were defined as *GZMA*, *GZMB*, *GZMH*, *GZMM*, *GZMK*, *GNLY*, *PRF1* and *CTSW*. The inflammatory gene set was defined as *CCL2*, *CCL3*, *CCL4*, *CCL5*, *CXCL10*, *CXCL9*, *IL1B*, *IL6*, *IL7*, *IL15* and *IL18*. The general stress gene set was defined as *BAG3*, *CALU*, *DNAJB1*, *DUSP1*, *EGR1*, *FOS*, *FOSB*, *HIF1A*, *HSP90AA1*, *HSP90AB1*, *HSP90B1*, *HSPA1A*, *HSPA1B*, *HSPA6*, *HSPB1*, *HSPH1*, *IER2*, *JUN*, *JUNB*, *NFKBIA*, *NFKBIZ*, *RGS2*, *SLC2A3*, *SOCS3*, *UBC*, *ZFAND2A*, *ZFP36* and *ZFP36L1*. Each score was calculated by AUCell, as described below.

A previous study developed a scoring system to identify immunologically active tumors, referred to as “hot tumors” (Foy et al., 2022). The hot tumor gene set includes *CCL19*, *CCR2*, *CCR4*, *CCR5*, *CD27*, *CD40LG, CD8A*, *CXCL10*, *CXCL11*, *CXCL13*, *CXCL9*, *CXCR3*, *CXCR6*, *FASLG*, *FGL2*, *GZMA*, *GZMH*, *IDO1*, *IFNG*, *IRF8*, *LAG3*, *LYZ*, *MS4A1*, *PDCD1*, *TBX21*, *TLR7* and *TLR8*. This score was calculated by ssGSEA in our bulk datasets, as described below.

The eight major cell types, NK and T cell populations we further subdivided were collected. We then used the FindMarkers function in Seurat to identify representative markers for these cell populations, selecting the top 20 markers with a p-value less than 0.05 (Supplementary Table S6).

### Calculation of signature score

For scRNA-seq data, we used the R package AUCell v1.26.0 (Aibar et al., 2017) to calculate the signature score of a specific gene set. We first built the ranked expression matrix using the AUCell_build Rankings function, and then calculated the AUC value using the AUCell_calc AUC function. Detailed gene sets used in this study were defined in the above section.

We used single-sample gene set enrichment analysis (ssGSEA) in GSVA v1.52.3 (Subramanian A et al., 2005) to score the expression of feature genes of all major populations we identified above. The parameters for the gsva function were set as mx.diff = T, method = ssgsea, and kcdf = Gaussian. The ssGSEA score was introduced to quantify the relative infiltration of cell populations in the tumor microenvironment based on bulk RNA-seq and microarray data. Additionally, the Hot score, based on a 27-gene expression signature above mentioned, was calculated to evaluate the immunologically active tumors in the same samples. Specifically, a Hot score greater than 0 was considered to indicate hot tumors, while a score less than 0 indicated cold tumors.

### Survival analysis

Survival analysis was performed using the survival method in the R packages survival v3.7.0 (Therneau, 2024) and survminer v0.5.0 (STHDA, 2016). The coxph function was used to construct both univariate and multivariate risk models for overall survival (OS), estimate the regression coefficients for each factor, and calculate the hazard ratios (HR) and p-values. After constructing the gene-based risk model, we used the vif function in car package to assess the multicollinearity of the candidate genes, ensuring that non-collinearity was maintained. The forest plot was generated using the forestplot. The surv_cutpoint function was used to select the optimal cutoff value, while the survfit function was applied to fit the survival model and calculate the p value of the log rank test. Finally, the ggsurvplot function was used to generate Kaplan-Meier (KM) survival curves. To evaluate the prognostic performance of the risk score, we performed time-dependent ROC analysis using the survivalROC v1.0.3.1 package (Patrick and Heagerty, 2000) with cut-off points at 365 (1-year), 1,095 (3-year), and 1828 (5-year) days.

### Therapeutic response prediction

Predicted half maximal inhibitory concentrations (IC50) of the common antitumor drugs in LUAD were calculated using the calcPhenotype in oncoPredict v1.2 R package (Maeser et al., 2021). The training dataset was selected GDSC2Data (built into the oncoPredict package), which includes 198 drugs and 805 training samples. All parameters were set to their default values.

### Clinical tissue collection

LUAD tissue samples were procured from patients diagnosed at the Fuyang People’s Hospital. The study excluded patients who had undergone any pre-surgical treatment and those with a history of other malignancies (see Supplementary Table S7 for details). All samples included both tumor and distant normal lung tissues. Prior to sample collection, all participants provided their written informed consent for the clinical application and research use of their tumor tissues.

### RT-qPCR

Total RNA was extracted from liquid nitrogen–ground tissues using TRIzol reagent (TaKaRa RNAiso Reagent 9,180). The tissue powder was mixed with 1 mL of TRIzol and incubated at room temperature for 5 min, followed by the addition of 200 μL of chloroform. After centrifugation at 12,000 × g for 15 min to separate the phases, the aqueous phase was mixed with an equal volume of isopropanol to precipitate RNA. The RNA was washed with 75% ethanol and dissolved in 20 μL of RNase-free water. The quality and purity of RNA were assessed using a NanoDrop 2000 spectrophotometer (Thermo Fisher Scientific) and 1.5% agarose gel electrophoresis. Subsequently, reverse transcription was performed using the PrimeScript™ RT Reagent Kit with gDNA Eraser (TaKaRa, RR047A). The specific procedure was as follows: genomic DNA was removed by incubation at 42°C for 2 min, followed by reverse transcription at 37°C for 15 min and termination of the reaction by heating at 85°C for 5 s. Real-time quantitative PCR was carried out using TB Green Premix Ex Taq™ (TaKaRa, RR420A) on a Bio-Rad CFX96 system. The PCR reaction mixture (20 μL) contained 10 μL of TB Green Premix Ex Taq II, 0.4 μL of forward primer (10 μmol/L), 0.4 μL of reverse primer (10 μmol/L), 1 μL of cDNA template, and 8.2 μL of RNase-free water. The internal reference primers used were β-actin. The PCR program was as follows: initial denaturation at 95°C for 30 s; followed by 45 cycles of 95°C for 5 s and 60°C for 30 s; and finally, melting curve analysis was performed. Primers for nine genes used in real-time PCR are listed in (Supplementary Table S8). The relative expression levels were calculated using the 2^(-ΔΔCt) method, and the experimental results were expressed as the mean ± standard deviation.

### Multiplex immunofluorescence staining

The method of multiplex immunofluorescence staining is as follows: Paraffin sections were placed in xylene I for 12 min, xylene II for 12 min, absolute ethanol I for 6 min, 95% ethanol for 6 min, and 85% ethanol for 6 min in turn, and washed with distilled water to complete dewaxing and rehydration. Subsequently, the sections were placed in EDTA repair solution (pH 8.0) for microwave antigen repair (medium heat for 8 min, stop heating for 8 min, medium-low heat for 7 min), and then cooled naturally. After washing with PBS (pH 7.4) for 5 min three times, 3% hydrogen peroxide was added to block endogenous peroxidase at room temperature for 25 min, followed by washing with PBS three times. The sections were blocked with 3% BSA at room temperature for more than 30 min. The primary antibody against CD8 diluted at 1:50 was added and incubated at 4°C overnight. After washing with PBS, the corresponding fluorescently labeled secondary antibody (haokebio, HKI0029, Alexa Fluor 488) was added and incubated at room temperature in the dark for 50 min. After washing with PBS, the Flare signal amplification reagent was added for 3–5 min to enhance the signal, and then washed with PBS three times. The above steps (starting from antigen repair) were repeated to incubate with the primary antibody and the corresponding labeled secondary antibody (haokebio, HKI0029, Alexa Fluor 594) to complete the second labeling. Finally, the nuclei were stained with DAPI at room temperature in the dark for 8 min, washed with PBS, and then mounted with an anti-fade mounting medium. The primary antibodies used are as follows: PTGDR (sanjingbio, Sj-AB11585), PTGDS (sanjingbio, Sj-AB16367), α-SMA (haokebio, HKA50033), CD56 (haokebio, 14255-AP), and CD8 (haokebio, I10361A).

### Statistical analysis

All statistical analyses were conducted using R software v4.1.1. Statistical analyses in this study included the Wilcoxon rank sum test and Student’s t-test, as described in the figure legends. Pearson correlation was applied to assess correlations between *PTGDR* and *PTGDS* expression levels in NK cells, while Spearman correlation was used to evaluate the relationship between gene expression levels and other factors in the heatmap. We used the plotROC v2.3.1 package to generate ROC curves for classifying tumors as hot or cold based on gene expression and the risk model. The area under the curve (AUC) was calculated to quantify the model’s ability to distinguish hot and cold tumors.

## Results

### Overall metabolic landscape of cells in LUAD

To elucidate the cellular and metabolic composition and crosstalk events of tumors and other relative tissues in LUAD, tumor lung (T:14), and normal lung (N:11, adjacent normal tissue), metastasis brain (M.brain:10), metastasis lymph node (M.LN:7), normal lymph node (N.LN:10) and pleural fluids (MPE: 5) were obtained from dataset GSE131907 (Kim et al., 2020), a dataset from 44 patients with treatment - naïve LUAD during endobronchial ultrasound/bronchoscopy biopsy or surgical resection. After filtering the scRNA - seq data to exclude damaged or dead cells and putative cell doublets, a total of 177,042 cell transcriptomes were retained for subsequent analysis. Following gene expression normalization for RNA reads count, gene numbers and Cell Cycle scores (see methods), we applied principal component analysis (PCA) based on top 2000 highly variably expressed genes across the sequenced cells. We used the Harmony algorithm (Korsunsky et al., 2019) to integrate data, effectively reducing batch effects without noticeable differences at the sample or cell cycle levels (Supplementary Figure S1A, B). We further employed the Harmony-corrected principal components to generate a unified UMAP embedding space and then performed graph-based clustering (Supplementary Figure S1C) and annotated each cluster with their respective markers (Supplementary Figure S2A, B). The cells were classified into ten major cell types (Figure 1B), including epithelial cells (Epi; n = 26,144), T cells (T; n = 56,674), B cells (B; n = 21,903), fibroblasts (Fibro; n = 4,430), plasmacytoid dendritic cells (pDC; n = 861), natural killer cells (NK; n = 24,489), myeloid cells excluding pDC and mast cells (Myeloid; n = 36,294), endothelial cells (Endo; n = 2,107), and oligodendrocytes which were derived from brain metastasis samples (Oligo; n = 655). All cell types marked by markers in Figure 1C. The grade of infiltration for each of these major cell types was different from tissue and tumor stage (Figures 1D–G). Obviously, among different tissues, tumor and metastasis locations with higher numbers of epithelial cells show fewer immune cells (e.g., NK cells), and this trend becomes more pronounced between early and advanced stages of tumors.

Cells from major cell types were extracted into the metabolite-estimated object, with over 6,000 cells from NK, Myeloid, T, Epi, and B cell types downsampled to 6,000. The abundance of 560 secretory metabolites across all cells was estimated using MEBOCOST (Zheng et al., 2022), with each metabolite present in more than 1% of cells in at least 1 cell type. To assess the overall metabolic differences across different cell types, we applied PCA based on top 200 highly variably metabolite across cells in the metabolite-estimated object. We further employed the 30 principal components to generate a unified UMAP embedding space and then performed graph-based clustering (Figure 2A) and colored cells by their types. We found that Myeloid, Fibro, Epi, Mast, pDC and Oligo cells exhibited distinct secretory metabolic patterns compared to Lymphoid cells. Furthermore, all of the metabolites, sensors, and metabolite-sensor signaling axes have been reported of 10 cell types in the TME (Supplementary Figure S4A–C; Supplementary Table S4). Metabolic cell-cell communication events that Myeloid, Fibro, Mast, and Oligo cells tend to act as senders, communicating with Endo, NK, Fibro, pDC, Epi and Myeloid cells (Figure 2B). We aimed to identify landmark metabolites for each cell type by analyzing metabolites specific to each type and extracting the top 20 involved in cell-cell communication. Five metabolites—prostaglandin D2 (PGD2), D-Mannose, Choline, L-Cysteine, and Cholesterol—were highlighted (Figures 2C, D; Supplementary Table S5). Further analysis revealed that the metabolic signaling axes among cell clusters include the following pairs: prostaglandin D2 ∼ *SLCO2A1*, prostaglandin D2 ∼ *PTGDR*, L-Cysteine ∼ *SLC43A2*, D-Mannose ∼ *SLC2A3*, Choline ∼ *SLC44A2*, Choline ∼ *SLC44A1*, Cholesterol ∼ *RORA*, Cholesterol ∼ *LDLR* and Cholesterol ∼ *CD36* (Figure 2E). Among these, Choline, L-Cysteine, and Cholesterol have been widely reported to play crucial roles in tumor occurrence and development (Song et al., 2024; Hua et al., 2020; Fan et al., 2023). Interestingly, PGD2 emerged as a noteworthy metabolite showing higher mean abundance observed in Mast, Oligo, Fibro, and pDC cells, which are rarely studied in oncology (Figure 2D). More specifically, PGD2 plays a pivotal role in signaling through its associated aixes, such as PGD2 ∼ *SLCO2A1* and PGD2 ∼ *PTGDR*, suggesting potential metabolic implications in tumorigenesis, tumor invasion and metastasis. Notably, PGD2 ∼ *SLCO2A1* is primarily mediated by sender cells to Endo cells, while PGD2 ∼ *PTGDR* is linked to interactions with NK cells (Figure 2E).

### Tumor-suppressive role of PGD2 and its signaling axis

**FIGURE 3:**
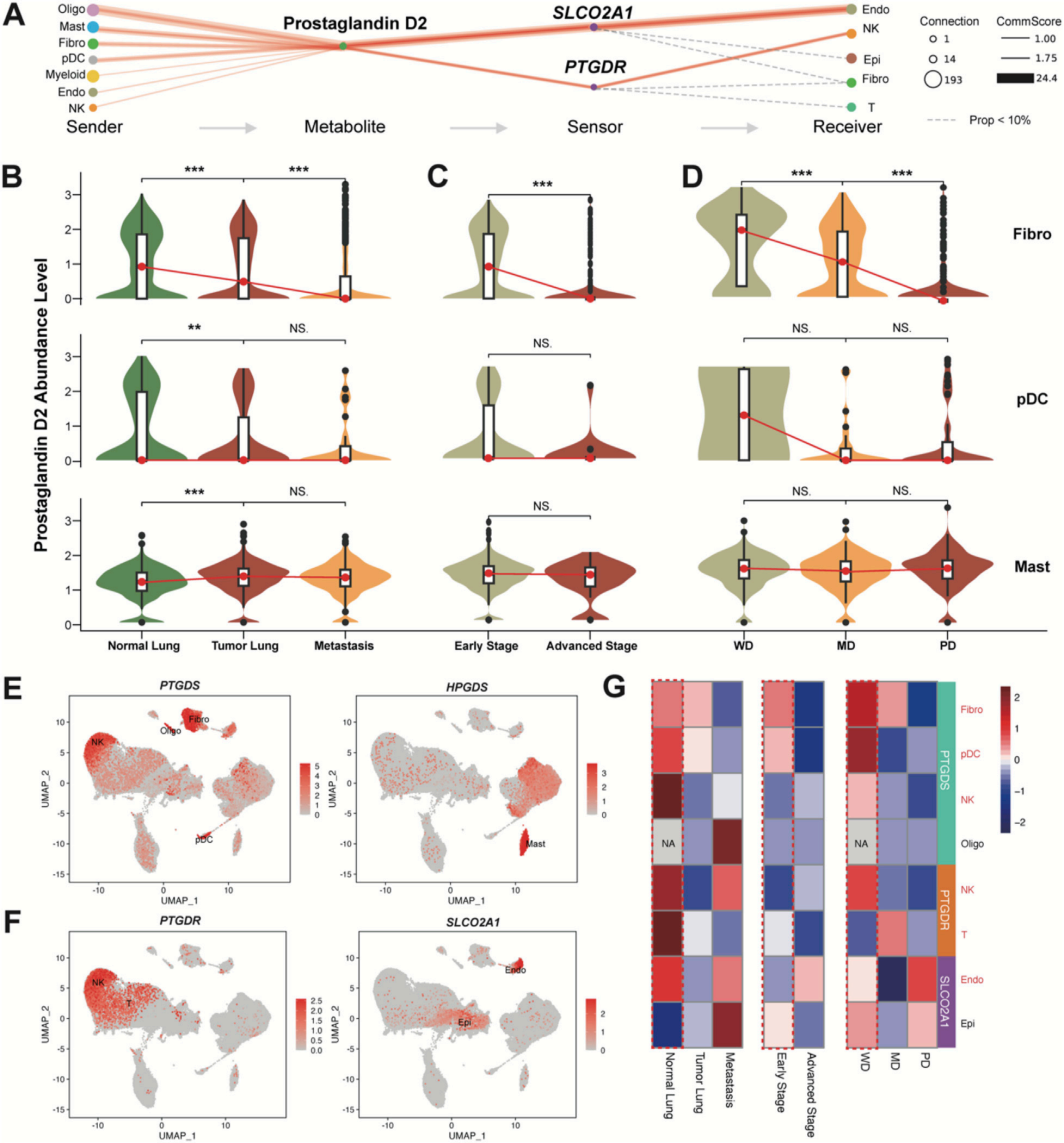
PGD2 and its enzymes and sensors levels. **(A)** Flow diagram showing the information flow of metabolite-sensor communications from sender cell type to receiver cell type through Prostaglandin D2 and its sensors SLCO2A1 and PTGDR. The size of dots represented the number of connections, indicating the frequency of usage among all the communications. The lines connect the sender, metabolite, sensor, and receiver. The solid red lines indicate communication scores >1, with the width of the lines representing the communication scores. The calculation follows the same method as the overall confidence shown in Figure 2E. The dashed gray line indicates the events that communication scores <1. **(B)** Violin plot of Prostaglandin D2 abundance levels in the top three sender cells (excluding Oligo) in Tumor Lung and Normal Lung). More detailed grouped expression patterns are shown in Supplementary Figure S5A. **(C)** Violin plot of Prostaglandin D2 abundance levels in the top three sender cells (excluding Oligo) in early stage and advanced stage. More detailed grouped expression patterns are shown in Supplementary Figure S5B. **(D)** Violin plot of Prostaglandin D2 abundance levels in the top three sender cells excluding Oligo) in different cell differentiation states. More detailed grouped expression patterns are shown in Supplementary Figure S5C. All significance levels, determined using the Wilcoxon test, is indicated by adjusted p-values: *p < 0.05, **p < 0.01, ***p < 0.001, and NS. > 0.05. **(E)** UMAP plot colored by the expression levels of enzyme genes *PTGDS* and *HPGDS.* **(F)** UMAP plot colored by the expression levels of sensor genes *PTGDR* and *SLCO2A1.* The rarely expressed *PTGDR2* gene is shown in Supplementary Figure S5D. **(G)** Heatmap of *PTGDS*, *PTGDR* and *SLCO2A1* expression levels across different groups (same as B-D) in top sender and receiver cells. The color represents expression (Z-score normalized mean expression level) in each cell type.

Focusing on PGD2, we further discovered that it is primarily produced in Oligo, Mast, Fibro, and pDC cells. Its metabolic signaling axes are primarily PGD2 ∼ *SLCO2A1*, which mediates communication prior to non-Lymphoid cells, and PGD2 ∼ *PTGDR*, which facilitates communication between Lymphoid cells (Figure 3A; Supplementary Table S9).

Notably, Oligo cells are present only in brain metastasis samples, so they will not be discussed further (Supplementary Figures S5A–C). In comparison, among the other three top PGD2-abundant cell types, the abundance of PGD2 shows a significant decreasing trend in Fibro cells and pDCs across normal lung, tumor lung, and metastatic brain tissues (Figure 3B), as well as in early and advanced stages (Figure 3C) and at different differentiation levels (well, middle, and poor; Figure 3D). Although the abundance of PGD2 is higher in mast cells, the associated trend is not as pronounced. *PTGDS* and *HPGDS* were signed as key emzymes for producing PGD2 in cells. We found that Mast, Fibro, pDC, and even NK/T cells produce PGD2 through *PTGDS*, while *HPGDS* is specifically expressed in Mast cells (Figure 3E). Additionally, *PTGDR* is specifically expressed in some NK/T cells, while *SLCO2A1* is primarily found in Endo and a small subset of Epi cells (Figure 3F), as inferred results from Figure 3A. The expression of *PTGDS*, *PTGDR* and *SLCO2A1* also shows a decreasing trend across normal lung, tumor lung, and metastatic brain tissues, as well as in early and advanced stages and at different differentiation levels. Based on these, we infer that PGD2 and its intercellular signaling axis play a tumor-suppressive role in LUAD.

To further validate this finding, we incorporated several publicly available integrated datasets from pan-cancer studies. We specifically extracted data related to LUAD or lung tissue, including normal samples, and confirmed that the cellular localization and disease progression relationships of *PTGDS*, *PTGDR*, *HPGDS* and *SLCO2A1* were consistent with our study (Supplementary Figure S6; Supplementary Figure S8; Supplementary Figure S9). These resources allowed us to cross-validate and enhance our findings, offering a broader perspective on the role of the PGD2 signaling axis in different cellular contexts within the tumor microenvironment.

### Function of PGD2 signaling axis in receiver cells

**FIGURE 4:**
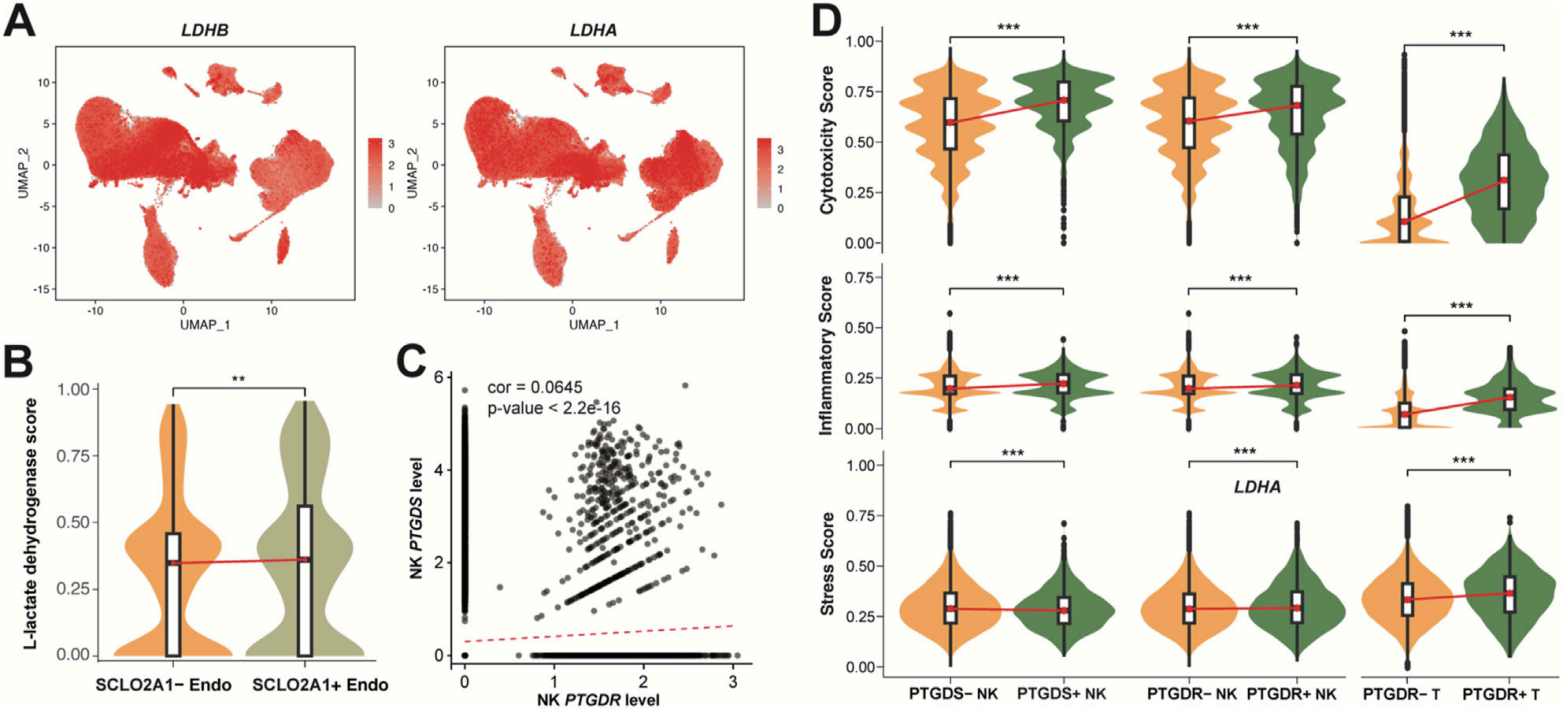
Correlation and functional role of Prostaglandin D2 in receiver cells. **(A)** UMAP plot colored by the expression levels of L-lactate dehydrogenase genes LDHB and LDHA. **(B)** Violin plot of L-lactate dehydrogenase score in the SCLO2A1^+/−^Endo. **(C)** Correlation between *PTGDS* and *PTGDR* expression in NK cells (Pearson correlation coefficient = 0.0645, p < 2.2e-16). **(D)** Violin plot of cytotoxicity, inflammatory, Stress score PTGDS^+/−^, PTGDR^+/−^NK and T cells. The scores in B and D represents the AUCell index of signature genes (see Methods). All significance levels, determined using the Wilcoxon test, is indicated by adjusted p-values: *p < 0.05, **p < 0.01, ***p < 0.001, and NS > 0.05.

To determine how PGD2 and relative signaling axis exert their tumor-suppressive function, we assessed the function of the sensors in the corresponding receiver cells. *SLCO2A1*, which encodes the solute carrier organic anion transporter family member 2A1 was a transporter of PGD2, facilitating its entry through lactate exchange (Lu et al., 1996). In our study, *LDHA* (encoding L-lactate dehydrogenase A chain) and *LDHB* (encoding L-lactate dehydrogenase B chain) are widely expressed in all cell types, including endothelial cells (Figure 4A). L-lactate dehydrogenase score for Endo cells base on *LDHA* and *LDHB* expression was calculated. We found that SLCO2A1+ Endo cells have a higher L-lactate dehydrogenase score, with statistical significance indicated by a p-value of (p < 0.05; Figure 4B). Therefore, we propose that the PGD2 ∼ *SLCO2A1* signaling axis may be associated with lactate efflux in Endo cells.

In NK cells, we observed that NK cell PGD2 autocrine signaling occurs, though potentially lower than paracrine signaling (Figure 3A). We therefore assessed the correlation of *PTGDS*, which encodes prostaglandin-H2 D-isomerase, and *PTGDR*, which encodes the PGD2 receptor protein 2, in NK cells, providing evidence that NK cells can mediate the PGD2 ∼ *PTGDR* process through autocrine signaling (Figure 4C). This weak correlation also suggests that the PGD2 ∼ *PTGDR* process in NK cells may simultaneously rely on paracrine pathways. We further examined the gene expression signatures to elucidate the impact of genes *PTGDS* and *PTGDR* on function of NK and T cells. Notably, PTGDS + NK cells, PTGDR + NK cells, and PTGDR + T cells exhibited significantly higher cytotoxicity and inflammatory scores, indicating their enhanced immune activity and implying a antitumor effects (Figure 4D). However, their influence on general cell stress was modest, as reflected by median stress scores, despite showing statistical significance.

### PGD2 signaling axis in NK and T cells

**FIGURE 5:**
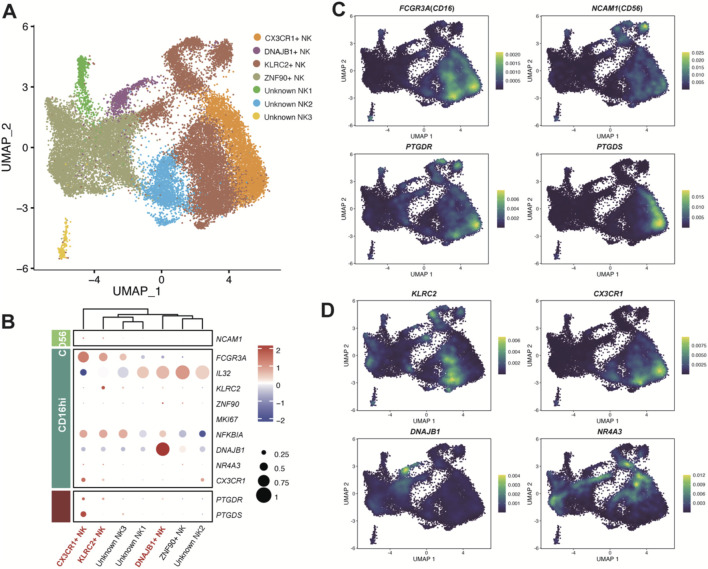
Enzyme gene *PTGDS* and receptor gene *PTGDR* observed in NK cells. **(A)** UMAP plot of NK cells colored by sub-clusters. Raw grouped clusters were presented in Supplementary Figure S7A. **(B)** Dot plot showing the expression pattern of marker genes for sub-clusters. The intensity of the color represents expression levels (Z-score normalized mean expression level), while the dot size indicates the percentage of cells in the respective sub-clusters. **(C)** UMAP plot colored by the expression level of *PTGDS*, *PTGDR,* and typical marker genes. **(D)** UMAP plot colored by the expression level of KLRC2, CX3CR1, DNAJB1, and NR4A3. Additional reference marker expression patterns can be found in Supplementary Figure S7C.

**FIGURE 6:**
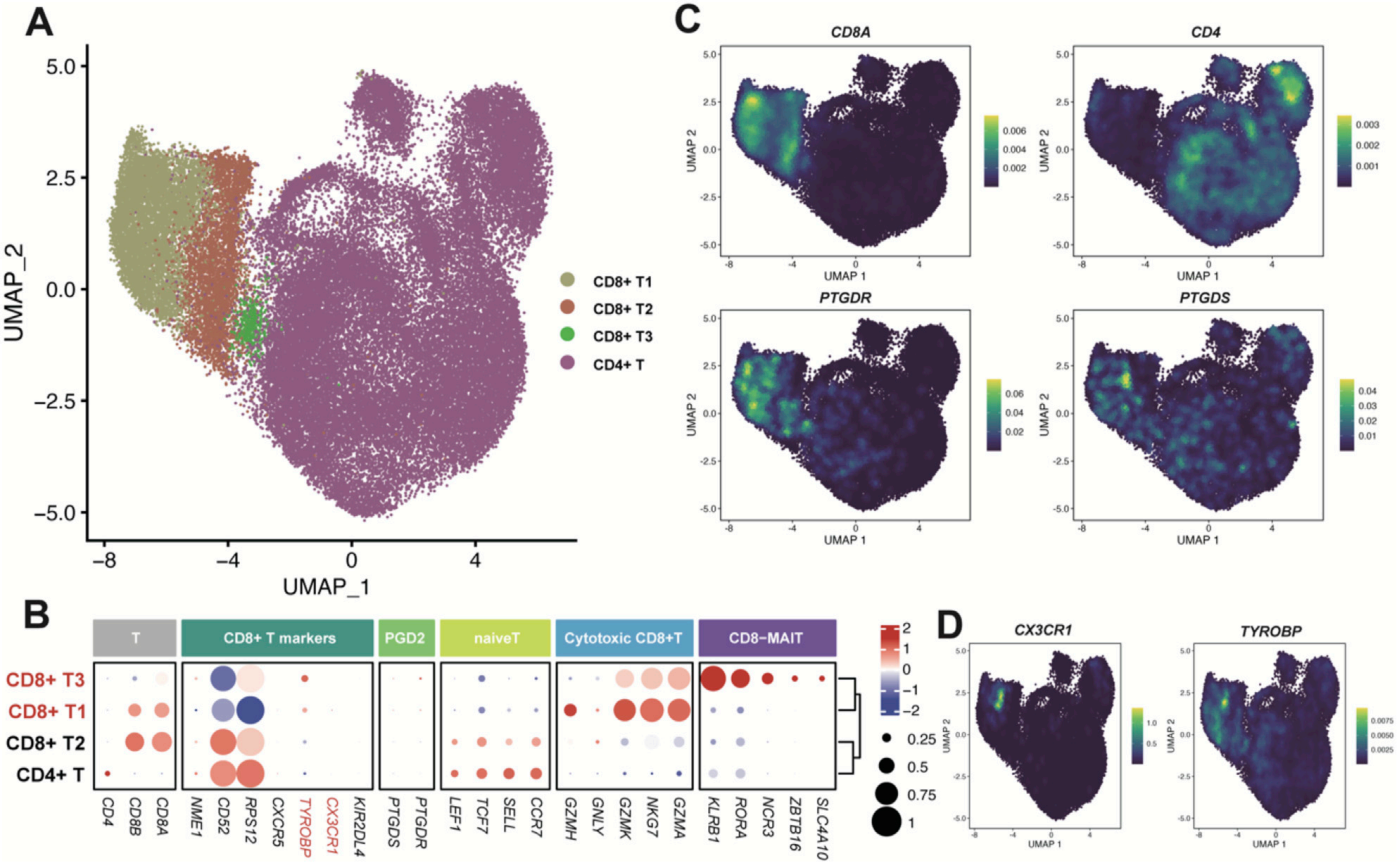
Enzyme gene PTGDS and receptor gene PTGDR observed in T cells. **(A)** UMAP plot of T cells colored by sub-clusters. **(B)** Dot plot for the expression pattern of marker genes of sub-clusters. The intensity of color represents Expression (Z-score normalized mean expression level); the dot size indicates the percentage of the respective sub-clusters. **(C, D)** UMAP plot colored by the expression level of *PTGDS*, *PTGDR* and typical marker genes.

We further deciphered the subpopulations of NK and T cells which participate PGD2 signaling axis aiming to identify more specific functional subpopulations (Figure 5; Figure 6), with raw clusters described in the classifications shown in Supplementary Figures S7A–C, 9A.

The NK cells were classified into six subpopulations (Figure 5A). As a previous study of pan-cancers NK cells reported that NK cells can be subdivided into two major populations, CD56^dim^CD16^hi^ and CD56^bright^CD16^lo^ (Tang et al., 2023). We considered PTGDS + NK cells in LUAD samples mainly belongs to CD56^dim^CD16^hi^, while PTGDR + NK cells are present in both populations, with a higher proportion in the CD56^dim^CD16^hi^ population (Figure 5B,C). Therefore, the PGD2 signaling axis seemed not to have a strong association with the distinction between CD56 and CD16 NK cell populations. Interestingly, CX3CR1+ NK cells exhibited high expression of both *PTGDS* and *PTGDR* (Figure 5B,C), suggesting a potential correlation between *CX3CR1* expression and the PGD2 autocrine signaling axis in NK cells. Meanwhile, DNAJB1+ NK cells and KLRC2+ NK cells predominantly expressed *PTGDR*, implying their association with the PGD2 paracrine signaling axis. Additionally, Unknown NK1, one of the subpopulations expressing *IL32* and likely involved in NK cell inflammatory responses, was also found to exhibit low-level expression of *PTGDR*. These gene expression correlations mentioned above were also validated in the online dataset (Supplementary Figure S8).

The T cells were classified into CD8+ and CD4+ T cells (Figure 6A), based on the high expression of canonical cell markers, CD8A and CD4 (Figures 6B,C). Since the genes *PTGDS* and *PTGDR* were mainly expressed in CD8+ T cells (Figure 6C), we further classified CD8+ T cells into three groups: CD8+ T1, CD8+ T2 and CD8+ T3. PTGDS and *PTGDR* highly expressed in CD8+ T1 and CD8+ T3 cells. CD8+ T1 cells tend to express cytotoxic markers such as *GZMA*, *GZMH*, *GZMK*, *GNMY*, and *NKG7*, while CD8+ T3 cells, which resemble mucosal-associated invariant T cells (MAIT), express markers as *SLC4A10*, *ZBTB16*, *NCR3*, *RORA* and *KLRB1*. Interestingly, both CD8+ T1 and CD8+ T3 cells highly express *TYROBP*, and CD8+ T1 also highly express *CX3CR1*, with the latter also co-expressed with *PTGDS* and *PTGDR* in NK cells (Figure 6D). These gene expression correlations mentioned above were also validated in the online CD8+ T cells dataset (Supplementary Figures S9B–D).

### Prognostic value of the PGD2 signaling axis

**FIGURE 7:**
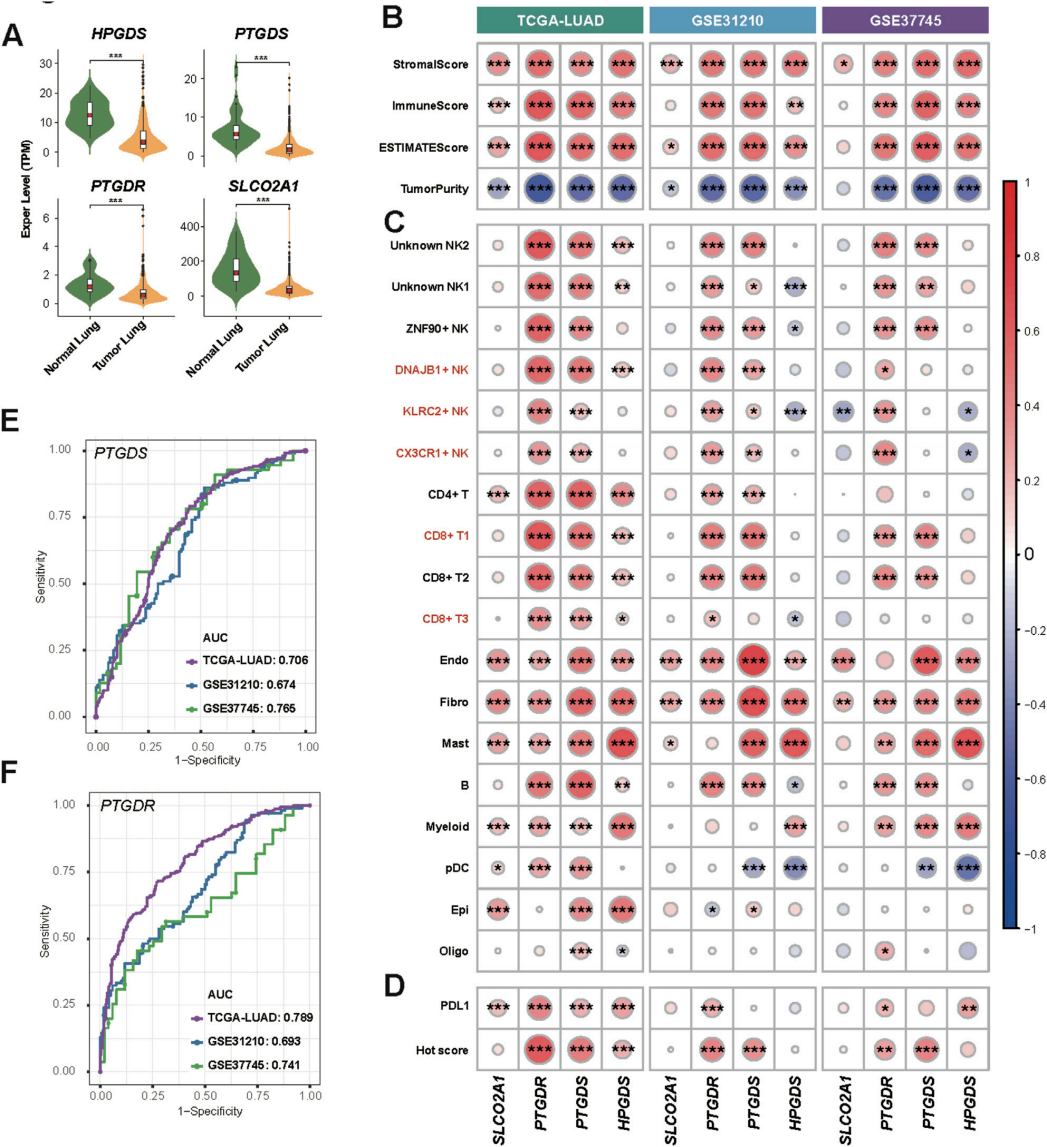
Enzyme and sensor genes of Prostaglandin D2 observed in bulk RNA data. **(A)** Violin plot of Enzyme and sensor genes expression of PGD2 in tumor lung and normal lung samples from the TCGA LUAD dataset. The Significance level, determined using the Wilcoxon test, is indicated by adjusted p-values: *p < 0.05, **p < 0.01, ***p < 0.001, and NS > 0.05. **(B)** Heatmap of Spearman correlation coefficients between genes and stromal, immune, and tumor scores in three bulk RNA datasets. **(C)** Heatmap of Spearman correlation coefficients between genes and cell type marker gene signature scores in three bulk RNA datasets. **(D)** Heatmap of Spearman correlation coefficients between genes and PDL1 and Hot scores in three bulk RNA datasets. **(E)** ROC plot showing the performance of *PTGDS* in distinguishing hot and cold tumors in three bulk RNA datasets. The AUC values are labeled separately for each dataset. **(F)** ROC plot showing the performance of *PTGDR*. ROC plot of *HPGDS* and *SLCO2A1* was in Supplementary Figures S10B, C. Details of score calculations are provided in the Methods. The significance level of Spearman correlation in **(B–D)** are indicated by p-values: *p < 0.05, **p < 0.01, ***p < 0.001.

We introduced three bulk RNA datasets (TCGA-LUAD, GSE31210, and GSE37745), which include more samples with clinical records, to explore the potential prognostic value of the PGD2 signaling axis. Firstly, relative genes involved in PGD2 signaling axis, *HPGDS*, *PTGDS*, *PTGDR* and *SLCO2A1*, were all significantly lower expressed in tumor lung tissue compared to normal in TCGA-LUAD cohort (Figure 7A). The expression differences of these genes across different stages, however, are not as pronounced, as shown in Supplementary Figure S10A. The StromalScore, ImmuneScore, and ESTIMATEScore were significantly positively correlated with these four genes, as determined by Spearman correlation analysis. In contrast, TumorPurity was significantly negatively correlated with these genes across all cohorts (Figure 7B). These findings validate the above results, suggesting that PGD2 cell-cell communication predominantly occurs between immune and stromal cells at the single-cell level and is associated with the degree of immune infiltration in tumors. Furthermore, we identified the top 20 marker genes for the major populations defined in this study and compiled them into custom gene sets (Supplementary Table S6). Based on these gene sets, we calculated the enrichment scores for each cell type in bulk RNA using ssGSEA and explore their correlation to relative genes involved in PGD2 signaling axis. We found that the enrichment of specific cell types corresponded closely with the expression of related genes. For example, the enrichment level of DNAJB1+ NK, KLRC2+ NK and CX3CR1+ NK cells were significantly associated with PTGDR gene expression (Figure 7C). To explore the clinical value of the PGD2 signaling axis, we calculated the Hot score for each sample across all cohorts using ssGSEA (Foy et al., 2022). These four genes were found to be significantly correlated with both the Hot score and PD-L1 expression levels, with *PTGDS* and *PTGDR* showing particularly strong associations (Figure 7D). This suggests that active PGD2 signaling axis activity may be associated with improved immunotherapy efficacy. The ROC plots further confirmed the predictive performance of *PTGDS* and *PTGDR* for distinguishing hot and cold tumors, with AUC scores ranging from 0.674 to 0.765 and 0.693 to 0.789, respectively (Figure 7E,F).

### Risk model based PGD2 signaling axis genes

**FIGURE 8:**
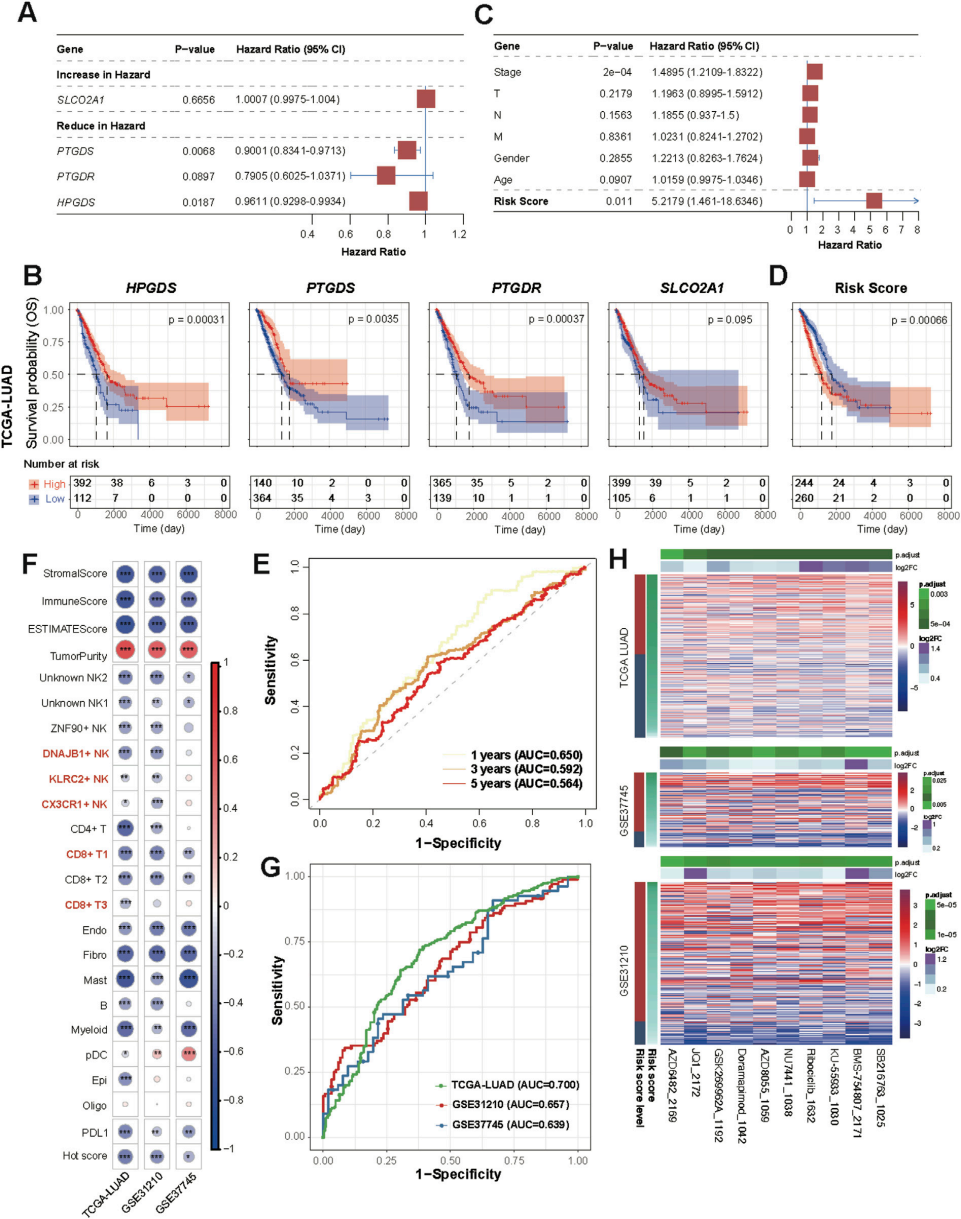
Clinical risk score based on Prostaglandin D2 enzyme and sensor genes. **(A)** Forest plot showing hazard ratios (HR) from the univariate Cox proportional hazards model, illustrating the association between individual variables and overall survival. HR values with 95% confidence intervals are shown, and statistically significant results are marked. **(B)** Kaplan–Meier survival curves for patients according to single gene expression (TCGA LUAD dataset). **(C)** Multivariate Cox proportional hazards model. Arrowhead is used to show that the lines extending from the box have reached the limits of the plot area. **(D)** Kaplan–Meier survival curves for patients according to Cox risk score (TCGA LUAD dataset). **(E)** Time-dependent ROC Curves at 1-year, 3-year, and 5-year Time Points. **(F)** Correlation between the risk score and all scores shown in Figures 7B–D. **(G)** ROC Curve of Risk Score for Classifying Hot and Cold Tumors. The AUC values are labeled separately for each dataset. **(H)** Heatmap of differential drug sensitivity across three datasets based on GDSC2 (198 drugs). The drugs were selected using the Wilcoxon test (p < 0.05) between high and low-risk groups based on Kaplan-Meier survival analysis.

Based on the TCGA-LUAD dataset, we performed univariate Cox regression risk scoring and for the genes involved in the PGD2 signaling axis, including *HPGDS*, *PTGDS*, *PTGDR* and *SLCO2A1* (Figure 8A). *PTGDS*, *PTGDR* and *HPGDS* were found to be associated with reduced risk, as indicated by their hazard ratios (HRs) of 0.9001 (95% CI: 0.8341–0.9713, p < 0.05), 0.7905 (95% CI: 0.6025–1.0371, p = 0.0897), and 0.9611 (95% CI: 0.9298–0.9934, p < 0.05), respectively. To evaluate the prognostic value of each gene, we performed Kaplan-Meier (KM) survival analysis for each of the four genes individually. Patients were stratified into high-expression and low-expression groups based on the optimal cut-off point of gene expression. The KM survival curves demonstrated that patients with higher expression levels of *PTGDS* (log-rank p < 0.001), *HPGDS* (log-rank p < 0.001), and *PTGDR* (log-rank p < 0.001) exhibited significantly better overall survival (OS) compared to those with lower expression levels of these genes (Figure 8B). This further supports their potential as prognostic bio-markers. Therefore, we established a multivariate Cox regression risk model based on these three genes, as follows: Risk score = PTGDS* (−0.07927835) + *PTGDR* * (−0.09647698) + *HPGDS* * (−0.0307662). *PTGDR*, *HPGDS*, and *PTGDS* had VIFs of 1.15, 1.05, and 1.20, respectively. The VIF (Variance Inflation Factor) values for all variables in the model were well below the threshold of 5, indicating no significant multicollinearity between them (Supplementary Table S10). Upon evaluation, the risk score derived from the model was found to be independent of clinical factors such as cancer stage, T (tumor size), N (lymph node involvement), M (metastasis), gender, and age (Figure 8C). Kaplan-Meier survival analysis stratified patients into high- and low-risk groups based on the optimal cut-off of risk score. The high-risk group exhibited significantly worse OS compared to the low-risk group (log-rank p < 0.001) (Figure 8D). The median survival time for the high-risk group was 1,194 days, while the low-risk group had a median survival time of 1,778 days, confirming the predictive power of the risk score in determining patient prognosis. Subsequently, the performance of these models was validated in the GSE31210 and GSE37745 datasets (Supplementary Figure S10D, E). The time-dependent ROC curves showed that the AUC remained modest across all time points, indicating that the model maintained a reasonable level of predictive performance over time (Figure 8E). Specifically, the AUC at 1 year was 0.650, at 3 years was 0.592, and at 5 years was 0.564. Although the model demonstrated some ability to discriminate between high-risk and low-risk patients, the decrease in AUC over time suggests that its discriminative power may diminish as the follow-up period extends.

We evaluated the correlation between the risk score, Hot score, and immune infiltration scores (e.g., ImmuneScore) in all cohorts (Figure 8F). Spearman correlation analysis revealed significant negative correlations between the risk score and immune infiltration scores, suggesting a potential link between higher risk and lower immune cell infiltration. Additionally, the risk score showed a significant positive correlation with TumorPurity, further supporting the association between immune response and tumor microenvironment. As expected, the ROC curves also demonstrated the ability of this model to differentiate between hot and cold tumors (Figure 8G). These findings align with the above results (Figures 7B–D), suggesting that genes involved in the PGD2 signaling axis exhibit protective factor properties and are positive correlated with immune infiltration.

Based on the 198-drugs GDSC2 database, we predicted the drug sensitivity of samples from TCGA-LUAD, GSE31210 and GSE37745 datasets. A differential test (Wilcoxon test, p < 0.05) was performed on the predicted IC50 values, stratified by high and low-risk groups based on Kaplan-Meier survival analysis. Ten drugs showed significant differences in sensitivity across the three datasets, which are as follows: SB216763, KU-55933, NU7441, Doramapimod, AZD8055, GSK269962A, Ribociclib, AZD6482, BMS-754807, and JQ1 (Figure 8H; Supplementary Table S11). Moreover, the predicted IC50 values for these drugs showed a decreasing trend as the risk score decreased, indicating that higher risk scores were associated with lower drug sensitivity. This further underscores that our PGD2 signaling axis risk model not only predicts prognostic risk but also facilitates the distinction between hot and cold tumors (which implies prognostic efficacy), and may provide valuable insights for treatment guidance regarding the ten drugs listed above.

### Validation expression levels of PTGDS and PTGDR in LUAD samples

**FIGURE 9:**
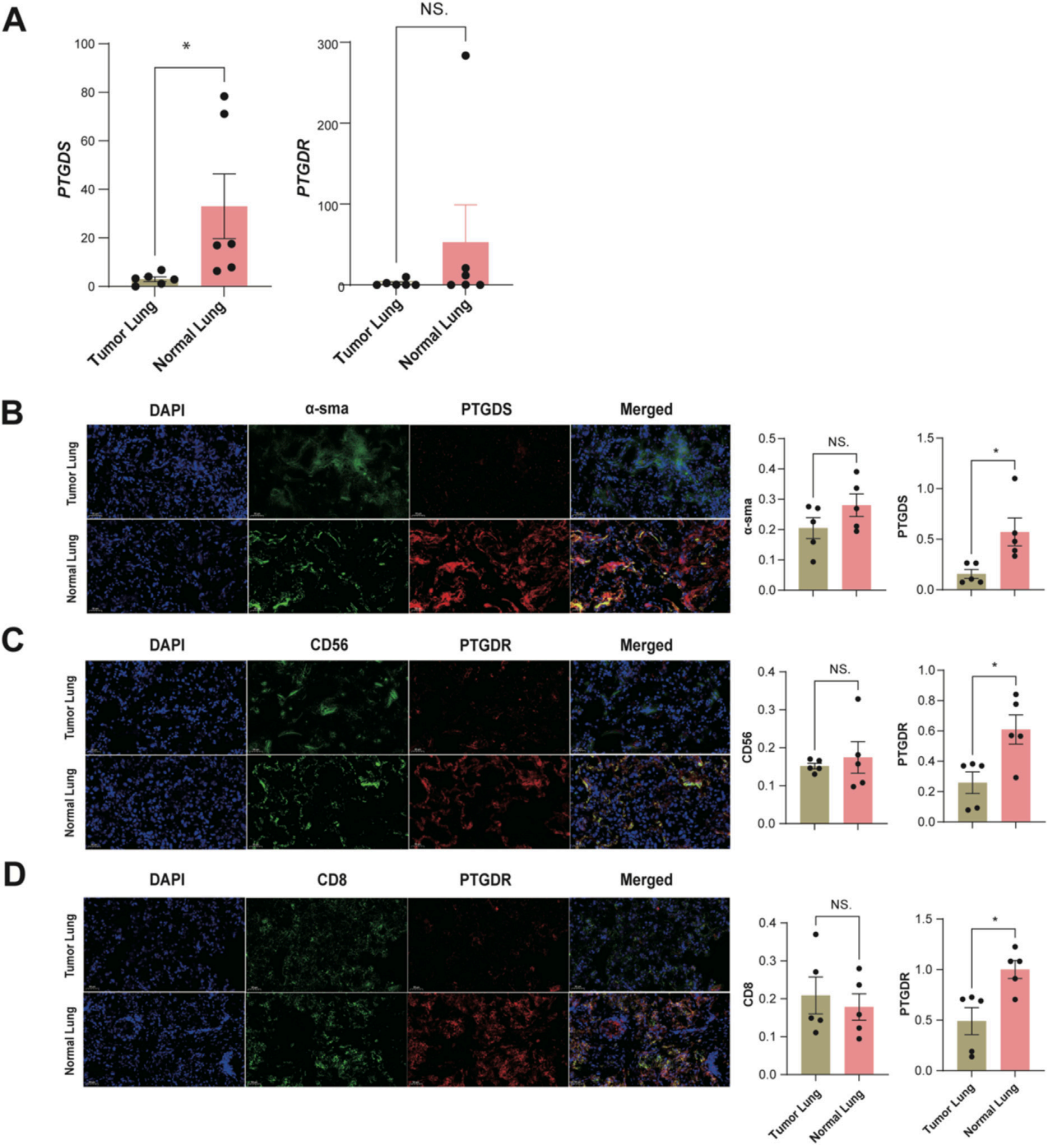
Preliminary experimental validation of PTGDS and PTGDR Characteristics in tumor and normal tissues (n = 5). **(A)** The mRNA relative expression levels in LUAD samples. Representative images of multiplex immunofluorescence staining for **(B)** α-sma + PTGDS + fibroblasts. **(C)** CD56+ PTGDR + NK cells. **(D)** CD8+ PTGDR + T cells. The relative fluorescence intensity for α-SMA and PTGDS in fibroblasts, CD56 and PTGDR in NK cells, and CD8 and PTGDR in T cells are presented to the right of the respective images. All significance levels, determined using the two-sided t-test, are indicated by adjusted p-values: *p < 0.05 and NS. > 0.05.

To confirm the expression patterns of PTGDS and PTGDR in tumor and normal tissues, we conducted a series of preliminary experiments using five pairs of LUAD tumor and distant normal lung tissues. The RT-qPCR results showed that *PTGDS* and *PTGDR* mRNA levels were lower in tumor tissues than in normal tissues (Figure 9A). PTGDS mRNA was significantly reduced (*p < 0.05), while PTGDR mRNA was reduced but not significantly (NS., p > 0.05). To further validate these two genes at the protein level, we performed multiplex immunofluorescence staining on tissue samples (Figures 9B–D). Similarly, we identified both CD56+ NK cells and CD8+ T cells co-expressing PTGDR. The relative fluorescence intensity of PTGDR in both CD56+ NK cells and CD8+ T cells was lower in tumor tissues compared to normal tissues (Figures 9C,D; p < 0.05). Collectively, these findings suggest that both PTGDS and PTGDR are downregulated in LUAD tumor tissues, with their expression patterns further substantiated in corresponding cell types.

## Discussion

Tumor metabolism and its role in the tumor microenvironment (TME) have drawn significant attention in cancer research (Mao et al., 2024; Nong et al., 2023; Park et al., 2020), yet the mechanisms of metabolite-mediated intercellular communication remain poorly understood. Advances in single-cell transcriptomic-based metabolism inference tools, now offer opportunities to explore these interactions. Building on this progress, our study investigates PGD2 as a key mediator of intercellular communication in LUAD, highlighting its signaling axis’s role in tumor immunity and therapeutic potential. Meanwhile, we also offered insights for future research on the discovery and validation of metabolite biomarkers.

Prostaglandins (PGs) are widely recognized as key mediators in cancer progression, with PGE2 often cited as a prototypical tumor-promoting marker (Santiso et al., 2024). In contrast, PGD2 distinguishes itself within the prostaglandin family through its tumor-suppressive properties, with PTGDS/PGD2 playing important roles in various cancer types and showing tissue specificity in function (Pan et al., 2021; Zhang et al., 2024; Shyu et al., 2013; Wu et al., 2012; Murata et al., 2011). Despite PGD2 being identified as a tumor-suppressive factor by numerous studies, most research has been based on *in vitro* experiments, with the underlying mechanisms remaining insufficiently explored (Iwanaga et al., 2014; Zhang et al., 2024; Omori et al., 2018). Additionally, most studies focus on the functional exploration of the PGD2/PTGDR2 receptor-ligand interactions (Tian et al., 2024). In lung cancer research, the tumor-suppressive role of PGD2 was proposed early on (McLemore et al., 1988). The vitro studies have shown that mast cell-derived PGD2 regulates the tumor microenvironment by limiting excessive responses to vascular permeability and TNF-α production (Murata et al., 2011). Previous studies have shown that L-PGDS (encoded by *PTGDS*) decreases proportionally with tumor progression (Ragolia et al., 2010). Furthermore, it has been demonstrated that exogenous L-PGDS can inhibit excessive proliferation and PDGF-stimulated migration of A549 cells. However, the full understanding of how PGD2 is produced and interacts with the LUAD immune microenvironment remains unclear. In this study, we identied and further explored the role of PGD2 and its intercellular signaling axis within TME of LUAD. Our findings suggest that PGD2 acts as a key metabolite, primarily produced by PGD2 synthase (*PTGDS*) in fibroblasts, mast cells and pDCs, influencing other cells through its signaling via the PGD2 receptor *PTGDR* on NK and T cells and the *SLCO2A1* transporter on endothelial cells. Finally, it is confirmed that the expression of PTGDS in fibroblasts and PTGDR in NK/T cells was downregulated at both the mRNA and protein levels in retrospectively collected paired tumor samples. Our study highlights the significance of PGD2 signaling, revealing its potential as a novel metabolic signaling axis that influences immune modulation within the TME of LUAD. These results reveal an important link between metabolic pathways and immune modulation in LUAD, offering new insights into the complexity of tumor-immune interactions.

In our analysis, we observed that the sender cells of PGD2 are primarily mast cells through HTGDS, which is consistent with previous studies (Murata et al., 2011). Furthermore, we identified other cell populations producing PGD2 through expressing *PTGDS*, including fibroblasts and pDCs and some populations of NK/T cells. Additionally, in brain metastasis patients, oligodendrocyte cells also produce PGD2 through the expression of *PTGDS*. On the other hand, studies on the receiver cells and sensors of PGD2 signals remain limited. We propose that CX3CR1+ NK/T cells may regulate cytotoxicity via the PGD2∼*PTGDR* autocrine pathway, contributing to antitumor effects. Meanwhile, KLRC2+ NK cells, DNAJB1+ NK cells and CD8^+^ MAIT cells participate in PGD2 paracrine signaling through PTGDR recepter. Although we hypothesize that PGD2 may also assist lactate efflux via SLCO2A1 on endothelial cells, this assumption lacks more direct evidence. Overall, these findings largely extends the current research on PGD2 signaling aixs in LUAD.

Previous studies have highlighted that PGD2 plays a crucial role in modulating immune responses (Joo and Sadikot, 2012). Our research further elucidates its regulatory signaling axis, at least in LUAD patients. A key finding is the involvement of CX3CR1+ NK/T cells in the PGD2-PTGDR autocrine pathway. *CX3CR1*, the receptor for *CX3CL1* (fractalkine), is expressed by various cell types such as CD8+ T cells and NK cells (Lysaght, 2020). Although myeloid cells expression of CX3CL1:CX3CR1 may be linked to tumor-promoting activities such as enhanced growth and migration in lung cancer (Okuma et al., 2017; Schmall et al., 2015), CX3CL1:CX3CR1 expression in bulk level is a positive prognostic factor in patients with LUAD (Liu et al., 2019). Our research highlights that CX3CR1+ NK/T cells, which exhibit strong cytotoxicity, are specifically involved in the PGD2 ∼ *PTGDR* autocrine pathway. This pathway maybe crucial for their immune functions and cytotoxic potential, underlining the complex role of PGD2 in LUAD tumor immunity.

In addition, we found receptor cells,KLRC2+ NK cells, DNAJB1+ NK cells and CD8^+^ MAIT cells, involved in PGD2 paracrine signaling pathway. According to other studiesthese 3 cell types also play anti-tumor roles within the tumor microenvironment. KLRC2+ NK cells are considered adaptive NK cells which lose the ability to kill autologous and activated immune cells (Schlums et al., 2015), while DNAJB1+ NK cells is a cell type with high stress scores (Tang et al., 2023). CD8^+^ MAIT cells have dual functions in promoting inflammation and mediating anti-tumor responses (Yigit et al., 2024). Overall, these receptor cells involved in paracrine signaling exert weaker tumor-killing effects than CX3CR1+ NK/T cells, and they may exhibit more complex and diverse regulatory. Our study support these, for example, the cytotoxicity-related gene expression in CD8+ T1 (CD8+ MAIT cells) is far weaker than in CD8+ T1 (CX3CR1+ T cells). Therefore, we infer that the self-secretory ability of PGD2 is strongly correlated with the cytotoxicity and tumor-killing capacity of CX3CR1+ NK/T cells. Additionally, some NK/T cells that lack self-secretory ability still express the *PTGDR* receptor, suggesting that other cells or exogenous PGD2 could enhance the anti-tumor activity of these cells in clinical settings.

Furthermore, the validation of PGD2’s role in LUAD using bulk RNA-seq datasets strengthens the generalizability of our findings. The presence of genes expression signatures across independent datasets suggests that PGD2 signaling axis might serve as a biomarker for immune-related responses in LUAD, providing a potential tool for both prognosis and therapeutic monitoring. As immune checkpoint inhibitors have shown limited success in certain subtypes of LUAD, strategies aimed at modulating PGD2 signaling could offer an alternative or complementary approach to overcoming immune resistance.

Our study has several limitations. Firstly, the estimation of metabolite abundance in our research is based on single-cell RNA expression, which is constrained by the limitations of current metabolomics technologies; further validation using metabolomics data could strengthen these findings. Secondly, the hypothesis of PGD2 coupling with lactate efflux need more robust experimental and data support. Lastly, our model did not exhibit outstanding performance across all validation datasets, likely due to batch effects in bulk RNA data.

In this study, we identified PGD2 as a critical metabolite within the LUAD tumor microenvironment, facilitating intercellular communication through its signaling axes, PGD2∼*SLCO2A1* and PGD2∼*PTGDR*. The analysis revealed that PGD2 mediates its tumor-suppressive effects by activating immune cells, particularly NK and T cells, which could be important for modulating anti-tumor immune responses. Our findings underscore the significance of PGD2 in regulating metabolic pathways and immune interactions in LUAD, highlighting its potential as a biomarker for prognosis and a target for future combination therapies. These findings highlight that PGD2 and its signaling axis contribute to tumor-suppressive and anti-inflammatory effects in LUAD tumor immunity, with potential applications in improving prognosis management and informing therapy decisions.

## Data Availability

The original contributions presented in the study are included in the article/Supplementary Material, further inquiries can be directed to the corresponding authors. The source code employed for data processing and analysis in this study is available in GitHub: https://github.com/zhengboying/PGD2_LUAD.
