# Supplementary figures and images for: Biological and prognostic insights into the prostaglandin D2 signaling axis in lung adenocarcinoma
Source: Front Pharmacol. 2025 May 22;16:1562261. doi: 10.3389/fphar.2025.1562261 (PMC12138261; doi:10.3389/fphar.2025.1562261)

FigS1

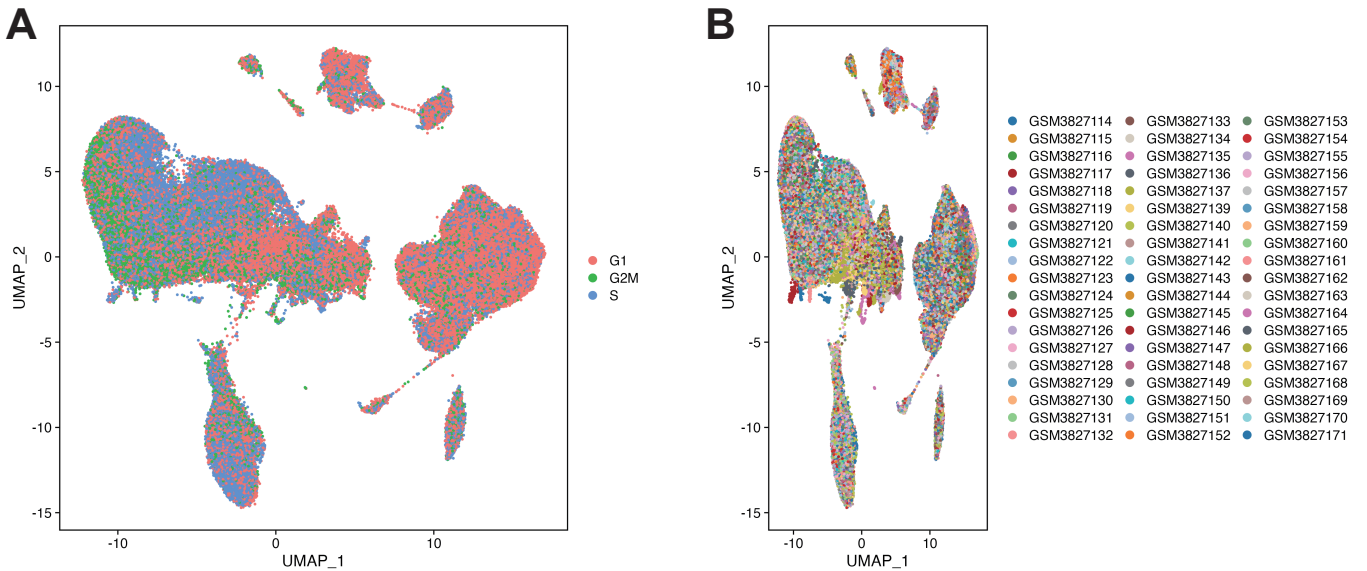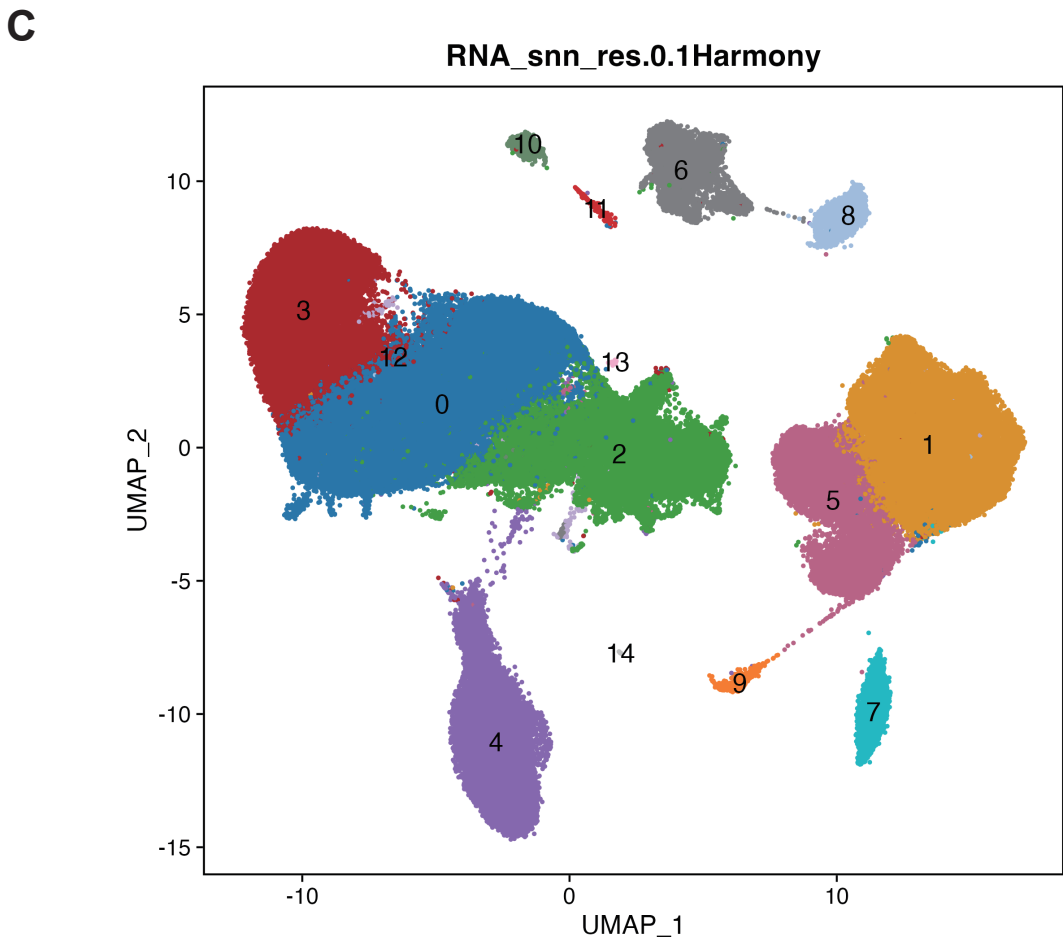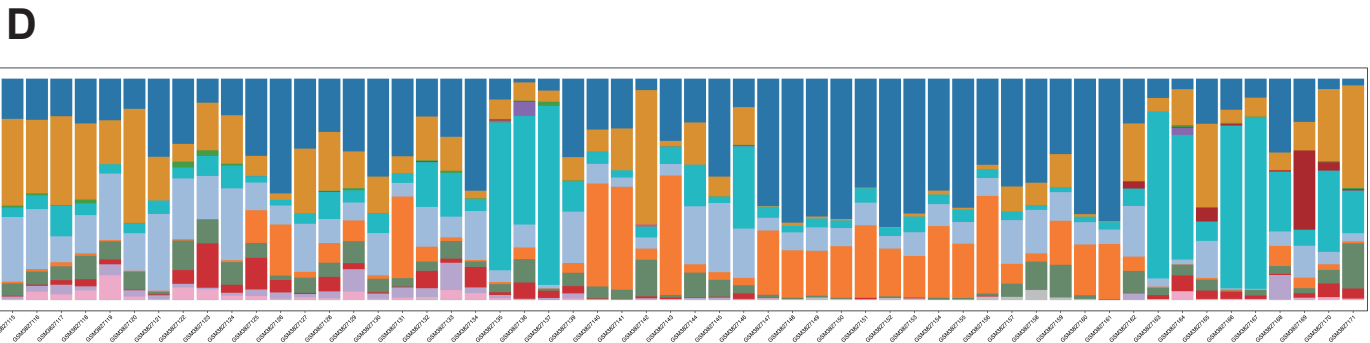

FigS2

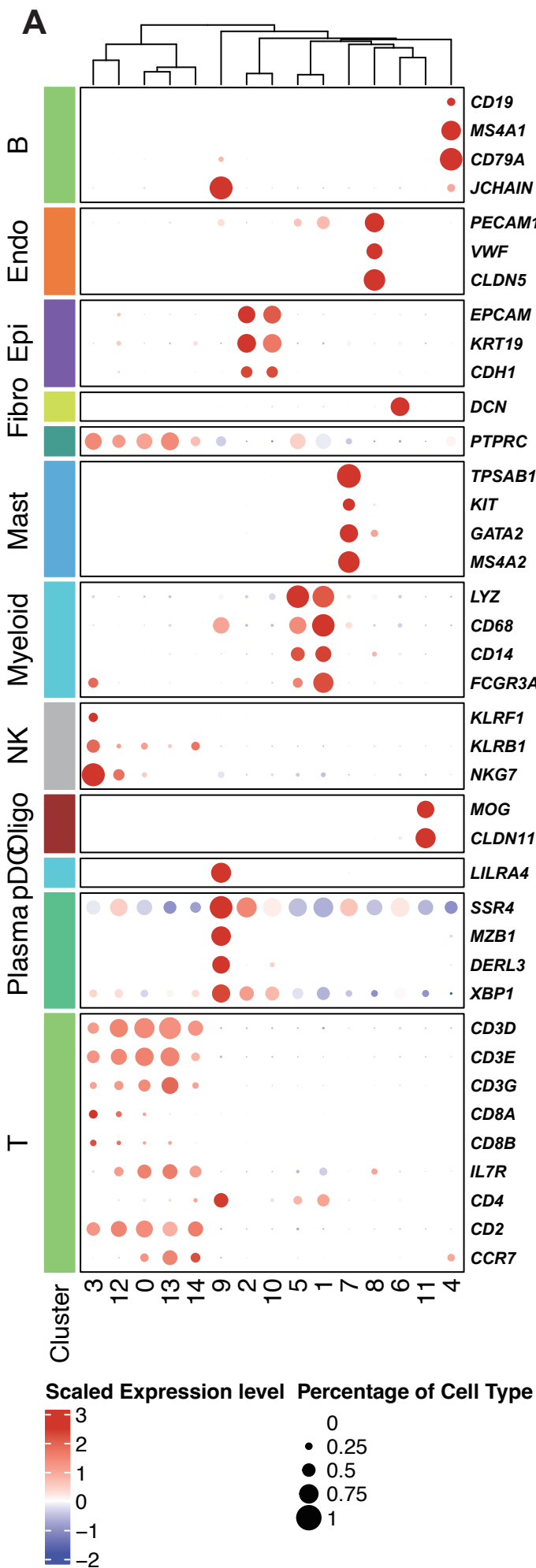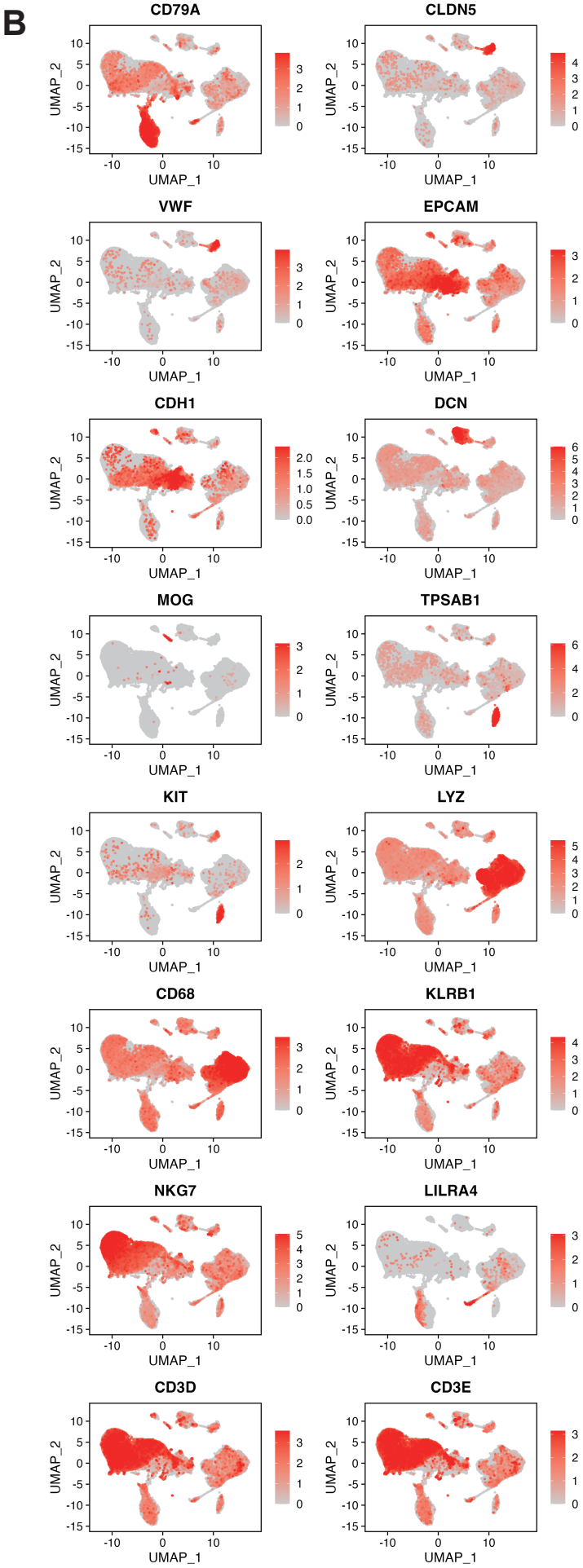

FigS3

A

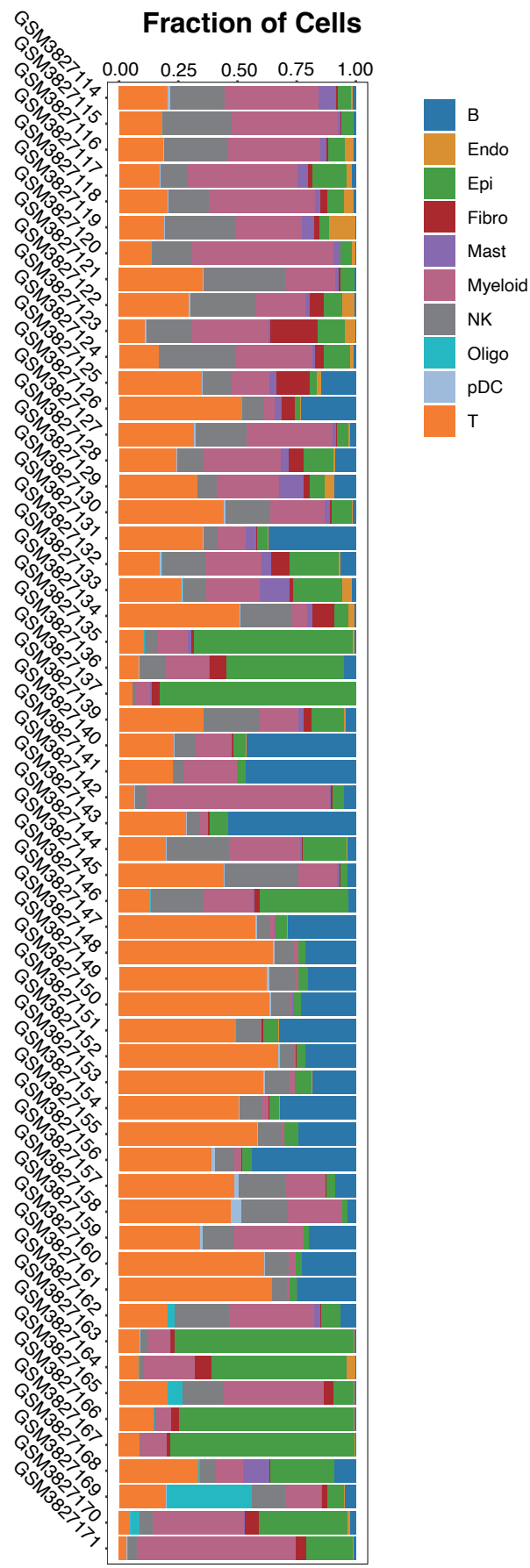

B

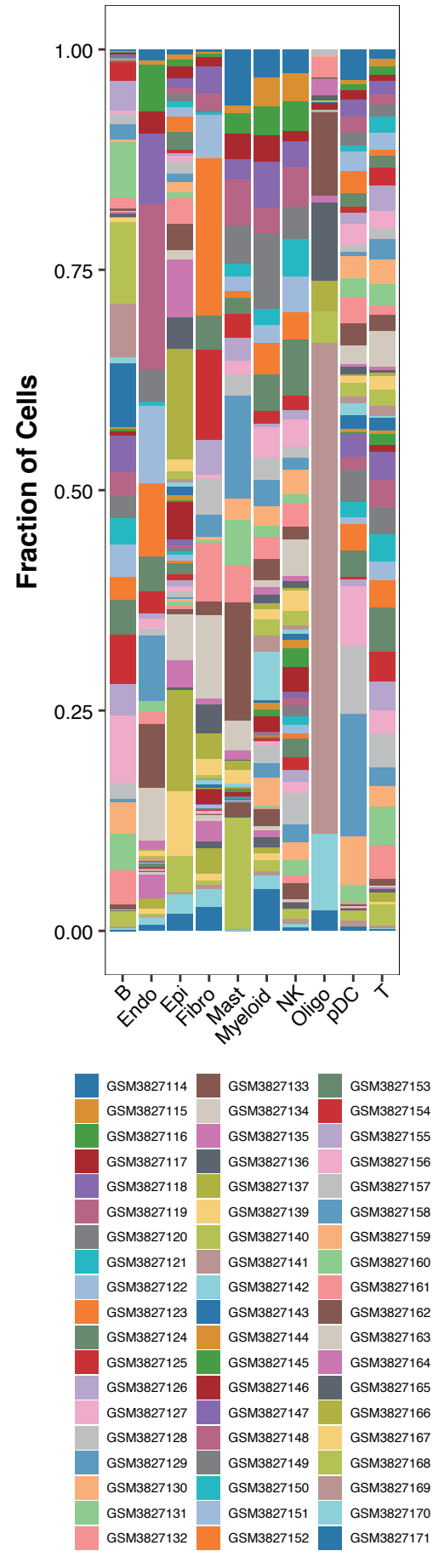

**A**

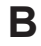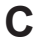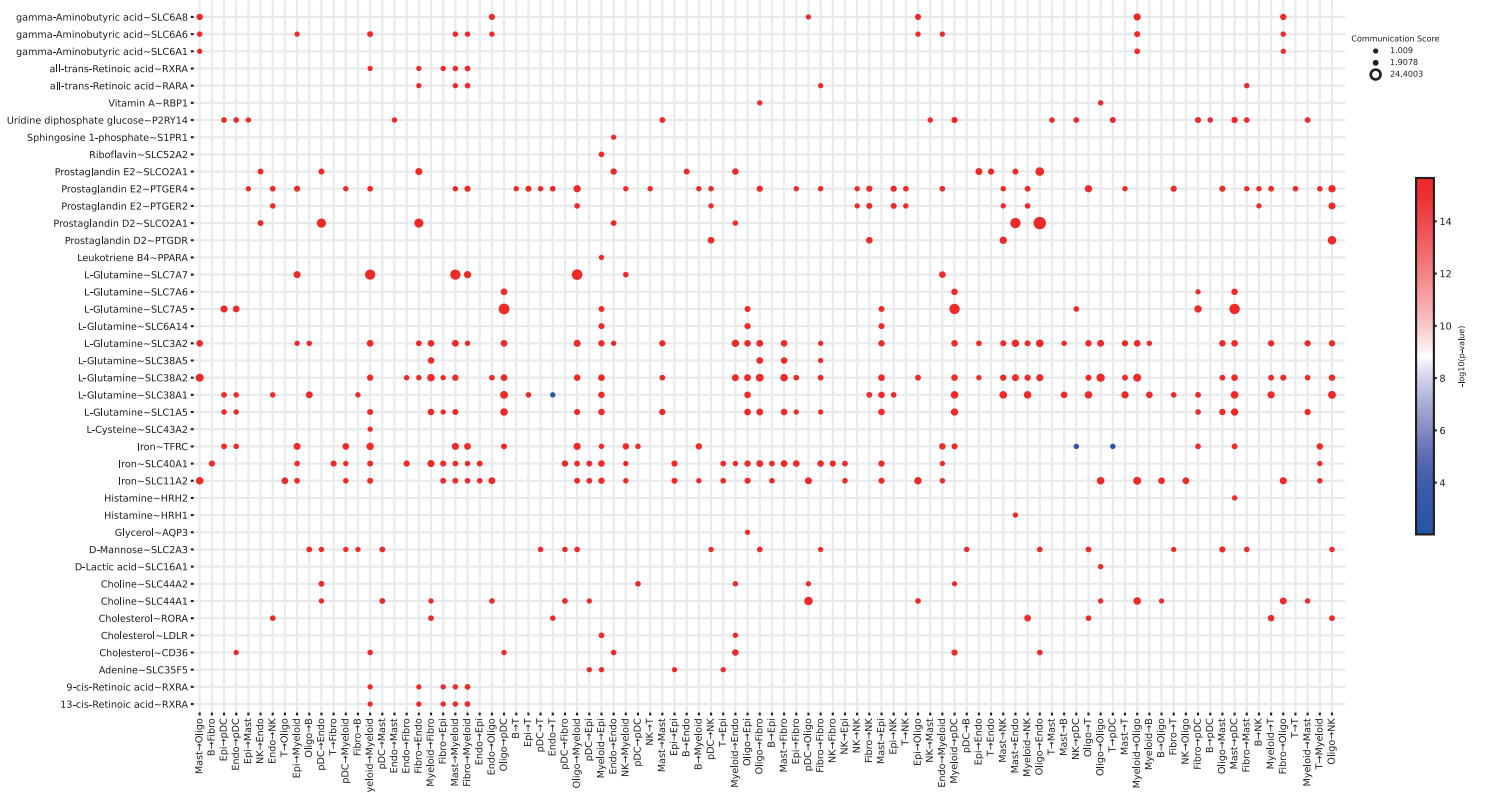

FigS5

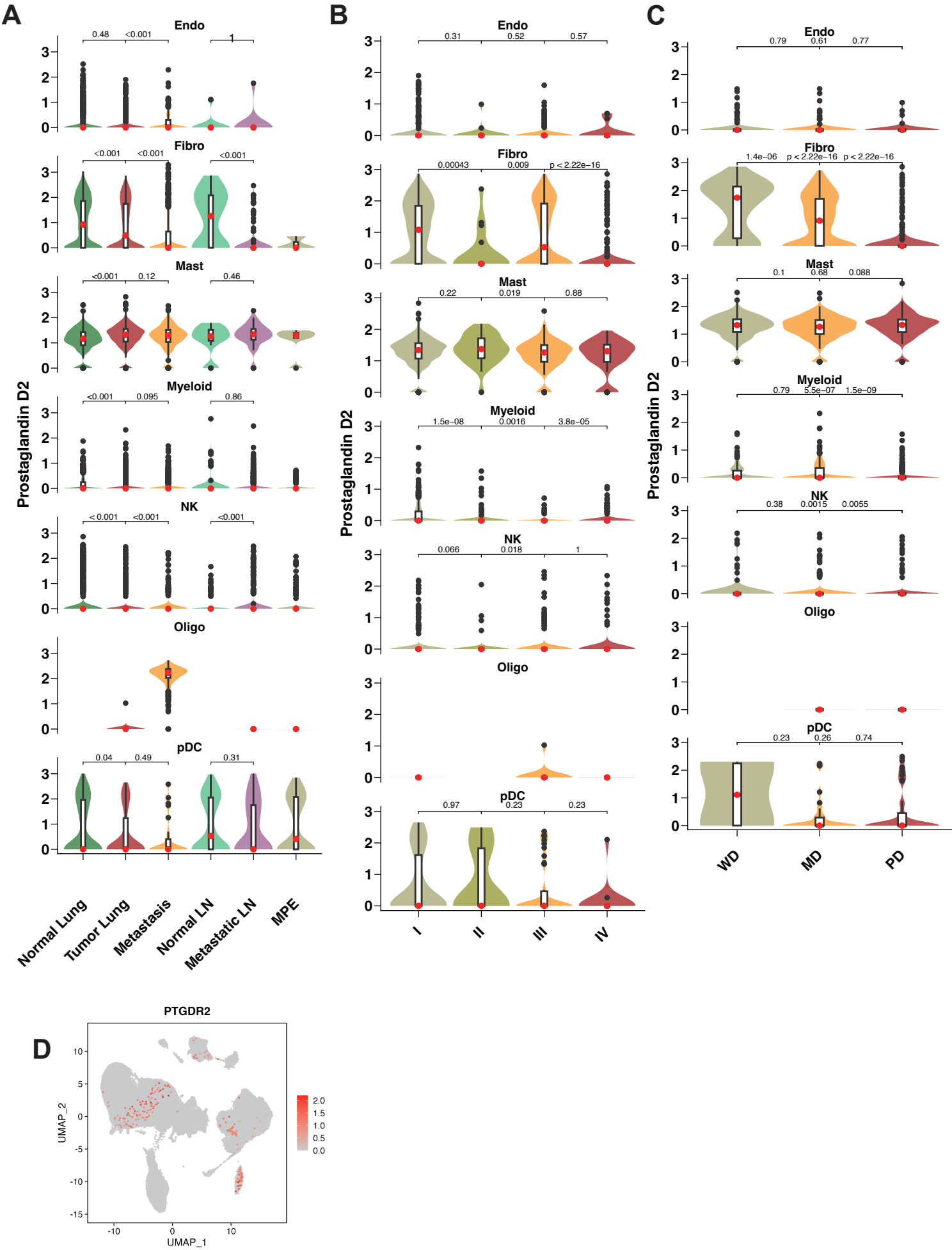

FigS6

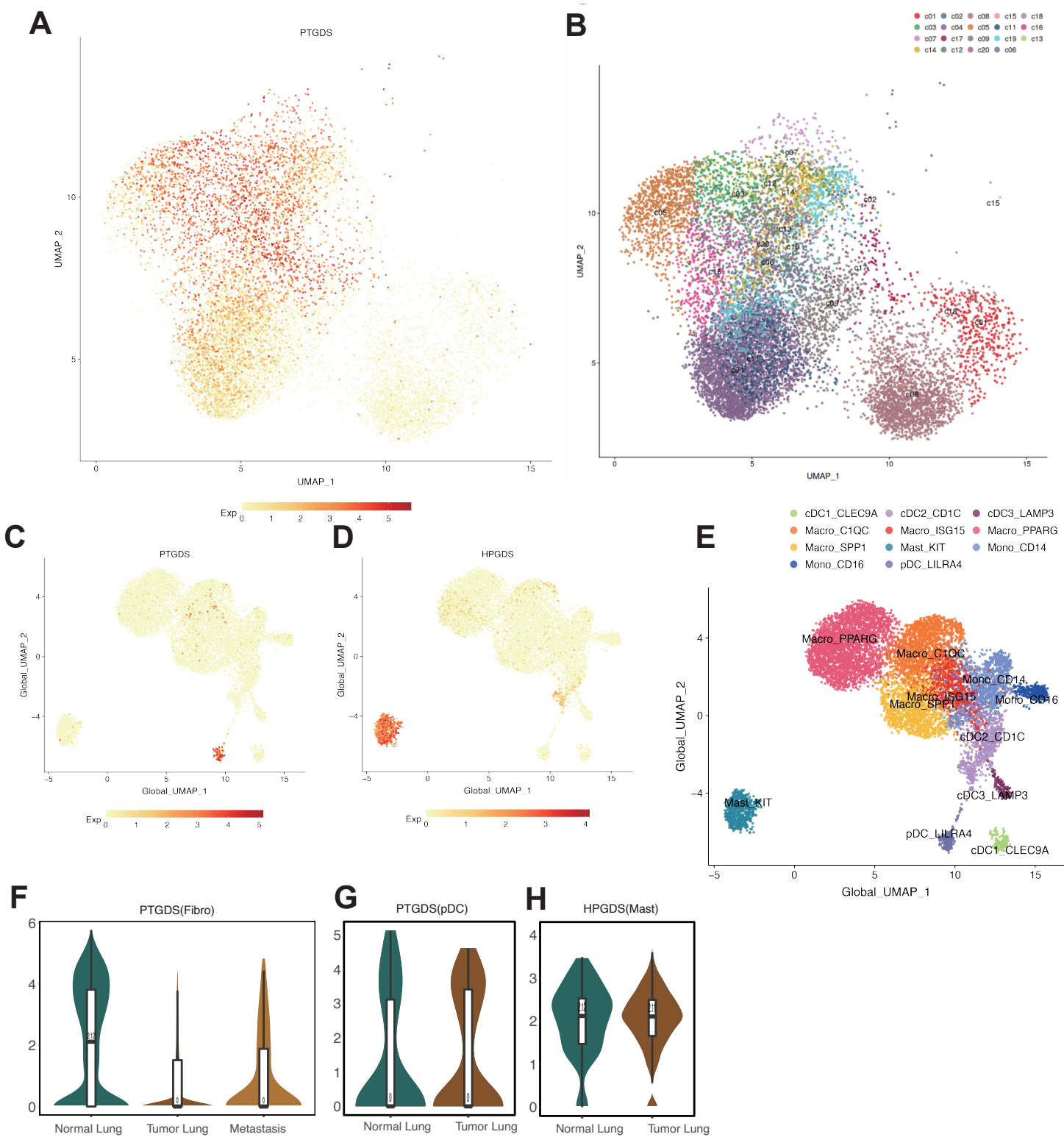

FigS7

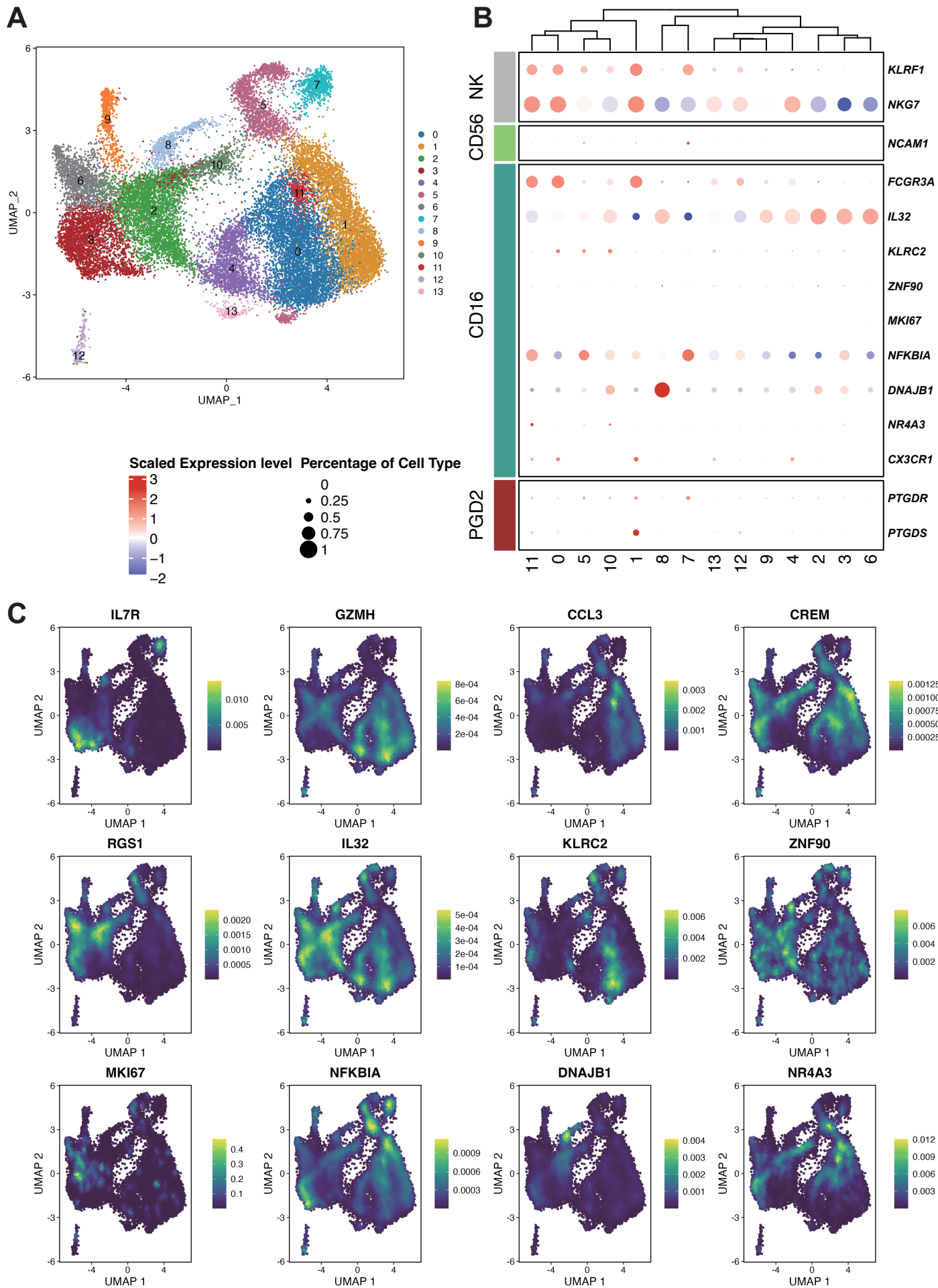

FigS8

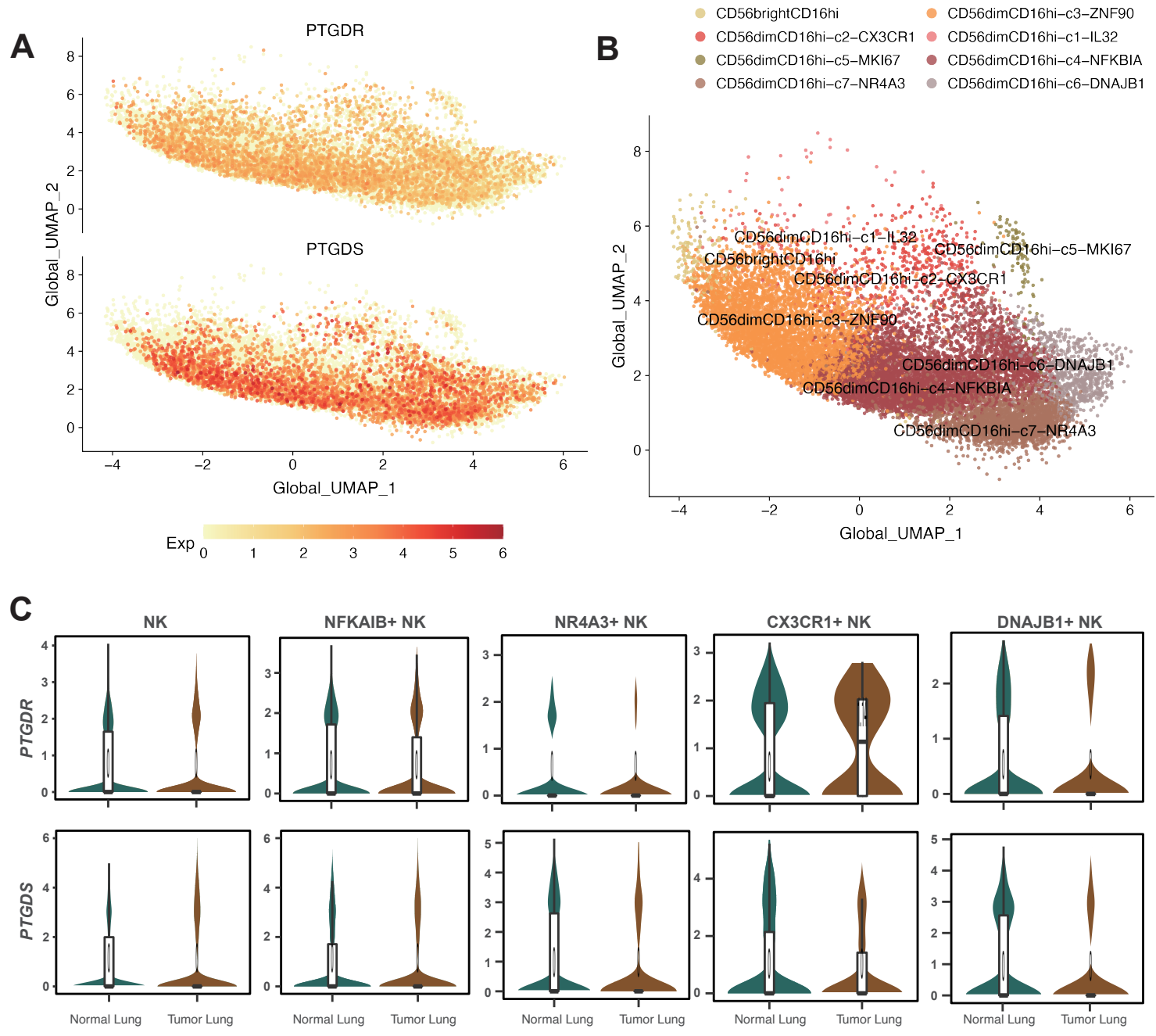

FigS9

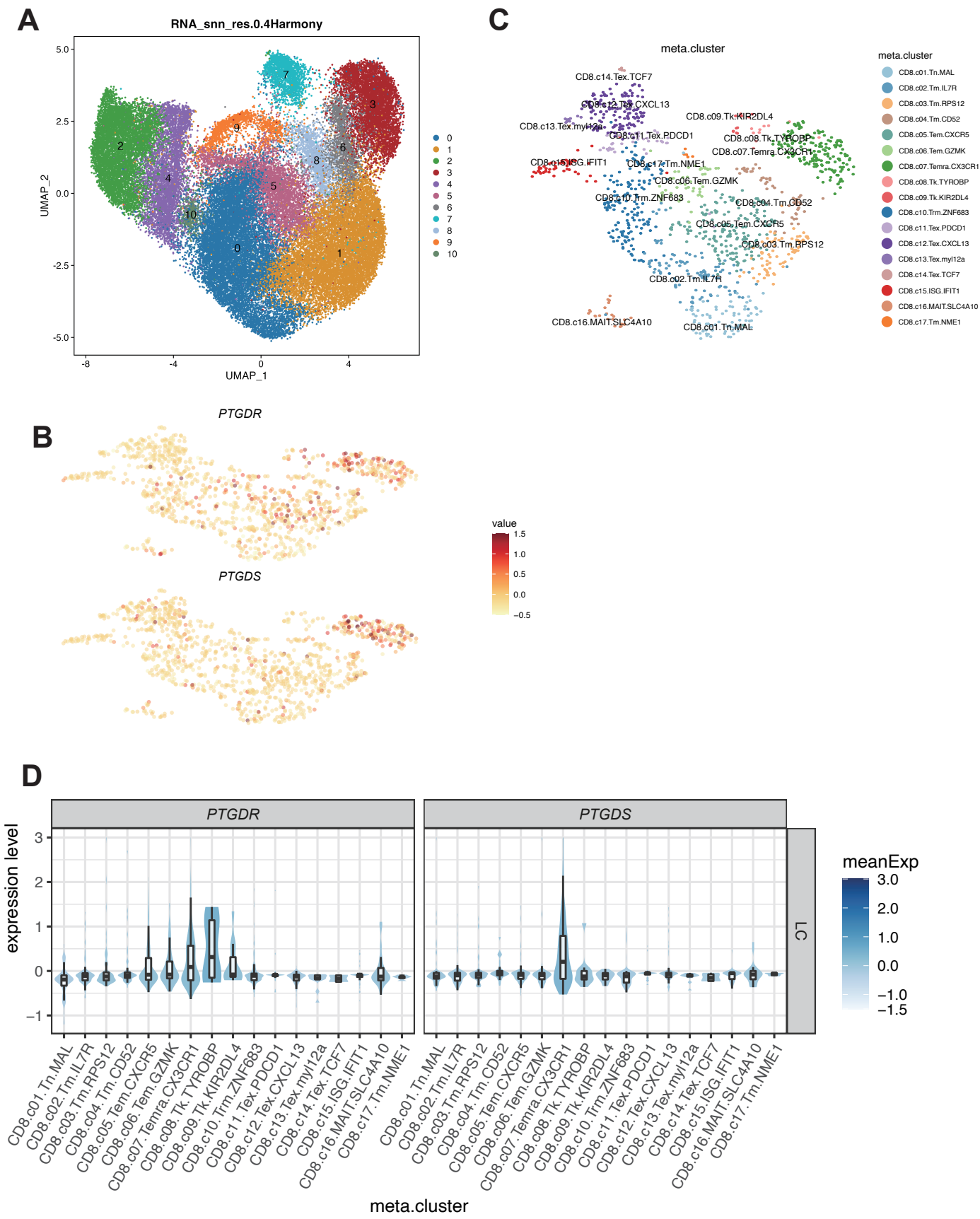

FigS10

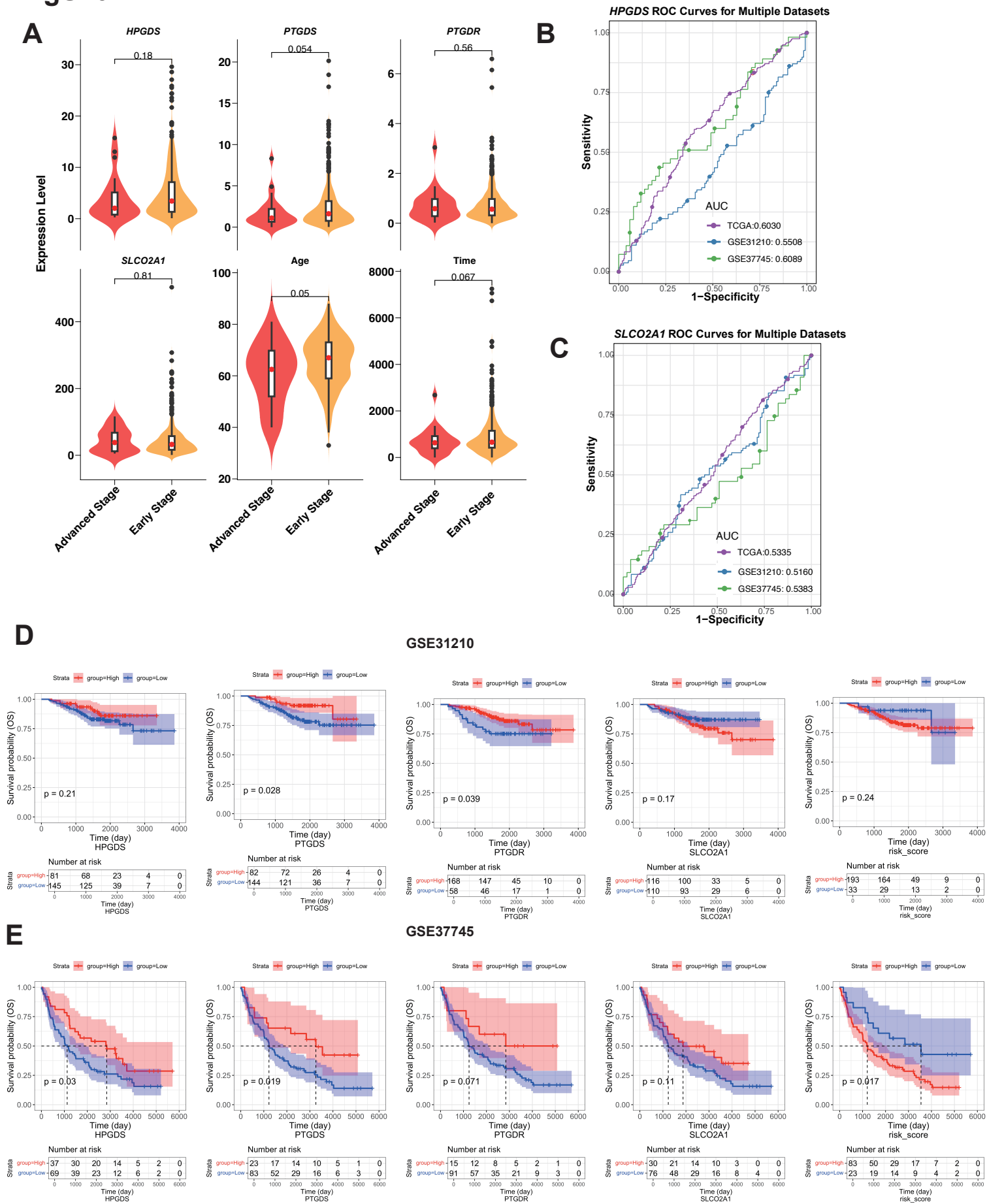

Supplement: Supplementary file 2 [file Image1.pdf]
